# Cold atmospheric plasma activated media selectively affects human head and neck cancer cell lines

**DOI:** 10.1111/odi.15120

**Published:** 2024-09-24

**Authors:** Viviana di Giacomo, Marwa Balaha, Morena Pinti, Maria Carmela Di Marcantonio, Ilaria Cela, Tirtha Raj Acharya, Nagendra Kumar Kaushik, Eun Ha Choi, Gabriella Mincione, Gianluca Sala, Miryam Perrucci, Marcello Locatelli, Vittoria Perrotti

**Affiliations:** ^1^ Department of Pharmacy “G. d'Annunzio” University of Chieti‐Pescara Chieti Italy; ^2^ UdA‐TechLab, Research Center “G. d'Annunzio” University of Chieti‐Pescara Chieti Italy; ^3^ Department of Pharmaceutical Chemistry, Faculty of Pharmacy Kafrelsheikh University Kafr El Sheikh Egypt; ^4^ Department of Innovative Technologies in Medicine and Dentistry “G. d'Annunzio” University of Chieti‐Pescara Chieti Italy; ^5^ Center for Advanced Studies and Technology (CAST) “G. d'Annunzio” University of Chieti‐Pescara Chieti Italy; ^6^ Plasma Bioscience Research Center, Department of Electrical and Biological Physics Kwangwoon University Seoul South Korea

**Keywords:** apoptosis, cell proliferation, cold atmospheric plasma, head and neck cancer, ionic chromatography, plasma activated media

## Abstract

**Objective:**

Cold atmospheric plasma (CAP) is a novel approach for cancer treatment. It can be used to treat liquids—plasma‐activated media (PAM)—which are then transferred to the target as an exogenous source of reactive oxygen and nitrogen species (RONS). The present study aimed at chemically characterizing different PAM and assessing their in vitro selectivity against head and neck cancer cells (HNC).

**Methods:**

PAM were obtained by exposing 2 and 5 mL of cell culture medium to CAP for 5, 10 and 20 min at a 6 mm working distance. Anions kinetics was evaluated by ion chromatography. Cell proliferation inhibition, apoptosis occurrence, and cell cycle modifications were assessed by MTS and flow cytometry, on human epidermal keratinocyte (HaCaT) and HNC cell lines HSC3, HSC4 and A253.

**Results:**

The 2 mL conditions showed a significant reduction in cell proliferation whereas for the 5 mL the effect was milder, but the time‐dependence was more evident. HaCaT were unaffected by the 5 mL PAM, indicating a selectivity for cancer cells.

**Conclusions:**

The media chemical composition modified by CAP exposure influenced cell proliferation by modulating cell cycle and inducing apoptosis in cancer cells, without affecting normal cells.

## 1 INTRODUCTION

Although significant efforts have been made to improve the detection and treatment of head and neck cancer (HNC), current therapies can only provide a 5‐year survival rate of up to 68% (Oral Cancer 5‐Year Survival Rates by Race, Gender and Stage of Diagnosis/National Institute of Dental and Craniofacial Research, 2018), which highlights the need to develop effective strategies. A limiting factor for a successful treatment is the extreme heterogeneity of this cancer, which might benefit from a multimodal therapy.

Cold atmospheric plasma (CAP) is a partially ionized gas made of physical and chemical components. It is well accepted that the main factors responsible for the anticancer properties of CAP are the reactive oxygen and nitrogen species (RONS) which are generated during the process and include hydrogen peroxide (H_2_O_2_), ozone (O_3_), hydroxyl radicals (•OH), superoxide (O_2_^−^), singlet oxygen (^1^O_2_), nitric oxide (NO), nitrite (NO_2_^−^), peroxynitrite (ONOO^−^), among others (Lin et al., 2019; Reuter et al., 2018). Other physical components (UV radiation, electric fields, visible light, etc.) have been shown to elicit a minor direct effect on biological samples and rather contribute to the formation of chemical species (Miebach et al., 2022). CAP targets cancer cells via apoptosis, necrosis, ferroptosis and immunogenic cell death, as reported in in vitro, in ovo and in vivo models (Kumar et al., 2021; Liedtke et al., 2018; Lin et al., 2019; Virard et al., 2015), with less harming effects on healthy cells (Biscop et al., 2019; Sperb et al., 2020). By oxidizing or destroying extracellular matrix (ECM) components, such as hyaluronan (Yusupov et al., 2021), CAP can inhibit tumor progression and it can improve drug delivery enhancing the sensitivity of cancer cells to chemotherapy drugs (Rasouli et al., 2021; Shaw et al., 2019, 2021). In addition, low doses of CAP‐generated RONS can have further positive effects since CAP can boost wound healing and decontamination (Bernhardt et al., 2019). Two types of plasma application modalities are currently used to treat cancer cells:

(i) Direct treatment involving direct exposure of the biological target to CAP (e.g. cancer cells in vitro; superficial tumours in vivo);

(ii) Indirect treatment involving the exposure of a liquid to CAP, and the subsequent application of these plasma‐activated media (PAM) onto the biological target, in vitro or in vivo, allowing minimally invasive therapy in the target site.

Direct CAP application has some limitations due to the narrow possibility of delivering RONS to internal target tissues. On the other hand, the indirect approach leads to the production of RONS more flexibly by the activation of different kinds of liquids with CAP and their administration as a treatment, regardless target's anatomic location (Kumar et al., 2018, 2021; Shaw et al., 2018, 2019, 2021). In this process, the CAP‐derived RONS are delivered from the plasma gas phase into the liquid phase, yet leaving a delicate mixture of long‐lived RONS, able to further recombine or react again to form intracellular short‐lived species (Bernhardt et al., 2019; Shaw et al., 2021). Some of the solutions used to generate plasma‐treated liquids (PTL) include water (Kumar et al., 2021), culture media (Azzariti et al., 2019), Ringer's lactate (Hashizume et al., 2021), phosphate buffer saline (PBS) solution (Mateu‐Sanz et al., 2020), Ringer's solution and bicarbonate Ringer's solution (Mateu‐Sanz et al., 2020).

The role of reactive oxygen species (ROS) in cancer pathophysiology is bivalent, since they can initiate the transformation of normal to malignant cells, but they have also a crucial effect on HNC progression (Aggarwal et al., 2019; Zhang et al., 2023). On the other hand, the increased production of ROS in cancer cells have them undergo under a higher oxidative stress compared to normal cells and can eventually lead to cell death. In addition, long‐lived species of PTL components (namely, H_2_O_2_ and NO_2_^−^) show strong synergy with tumor suppressor enzymes, which are located on the cell membrane. Indeed, ONOO—which is produced from H_2_O_2_ and NO_2_^−^, followed by the primary ^1^O_2_ causes inactivation of membrane‐associated catalase (Privat‐Maldonado et al., 2018; Shaw et al., 2021). Other reactive species can also be derived from the solutes of PTL (Van Boxem et al., 2017). Specifically, a nuclear magnetic resonance spectroscopy (NMR) analysis showed that acetyl‐ and pyruvic acid‐like groups are generated in Ringer's lactate solution treated by CAP, which has shown a crucial antitumor role (Tanaka et al., 2016). However, to effectively use PTL in the clinic, a thorough understanding of the interactions of RONS with biomolecules (lipids, proteins and nucleic acids) from the atomic to the macro scale, and their biological significance, is needed. Recent studies have shown that pro‐oxidant compounds can activate the protective antioxidant response in healthy cells while killing cancer cells, as the excess of RONS can quickly exhaust the antioxidant response available (Chaiswing et al., 2018; Du et al., 2015; Shin et al., 2020; Sznarkowska et al., 2017). Even more, it has been suggested that increased levels of RONS could sensitize cancer cells to other therapeutic approaches, such as chemotherapeutic drugs and radiotherapy (Alexander et al., 2019; Du et al., 2015). These findings highlight the potential clinical application of pro‐oxidant therapies; therefore, novel treatments that rely on the production and delivery of reactive species, such as CAP, arise as an interesting option for cancer treatment.

This study aimed to chemically characterize a set of PAM by a novel approach based on ion chromatography and to define the role of the chemicals generated in the newly developed PAM in relation to their biological effects on the inhibition of proliferation, apoptosis occurrence and regulation of cell cycle on a panel of HNC cell lines, in order to choose the best PAM with the potential to exert selective and comprehensive therapeutic effects towards HNC cells for future in vivo studies.

## 2 MATERIALS AND METHODS

### 2.1 Plasma system, diagnostics and RONS measurement technique

**FIGURE 1:**
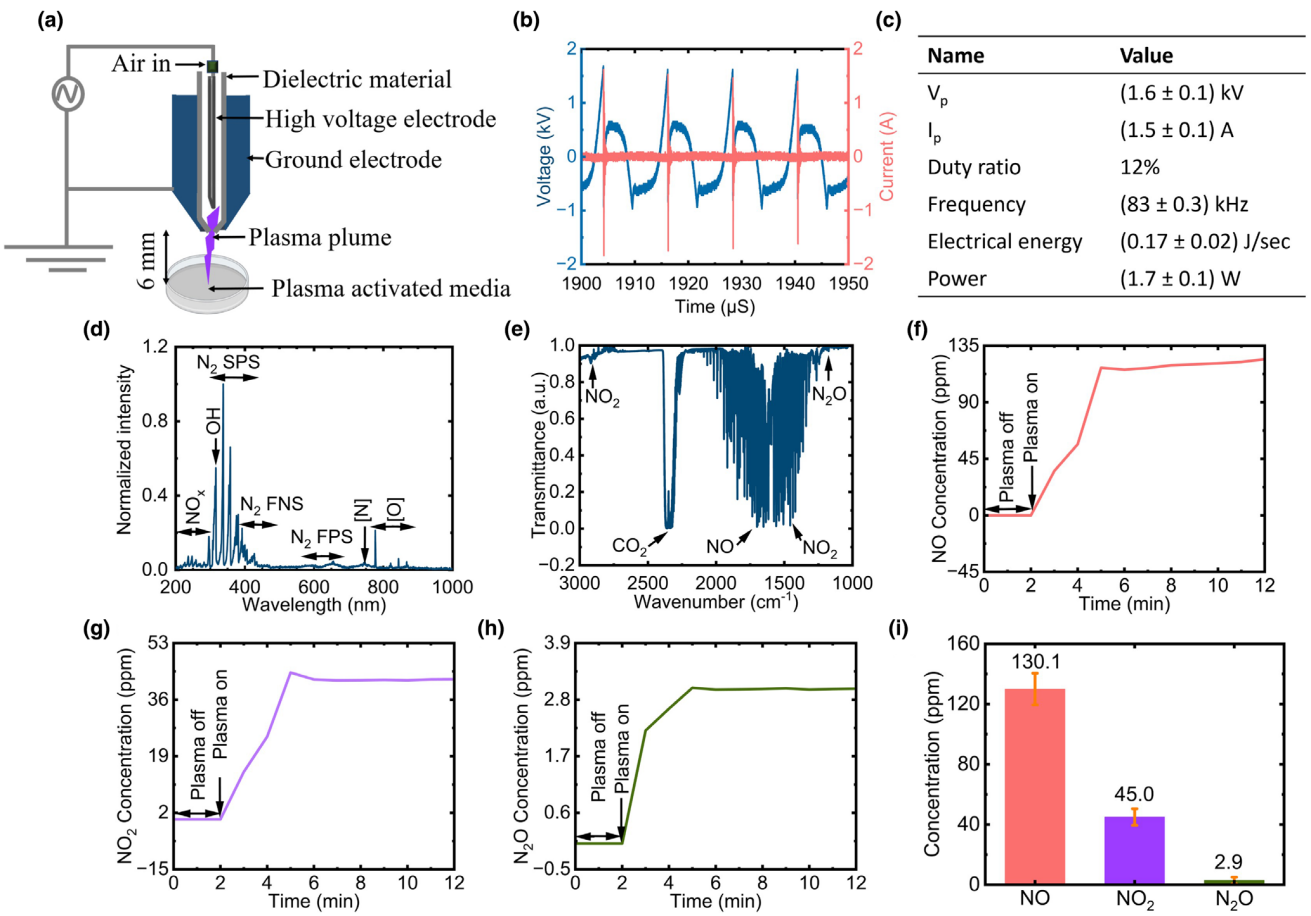
Plasma system and diagnostic analysis and physicochemical properties. (a) Illustration of the soft jet plasma setup; (b) current–voltage characteristics; (c) assessment of electrical properties within the soft jet plasma; (d) optical emission spectra (OES) demonstrating the presence of diverse reactive oxygen and nitrogen species (RONS); (e) gas Fourier transform infrared (FTIR) analysis of plasma‐generated reactive species; Quantitative determination of (f) NO; (g) NO_2_; (h) N_2_O concentration; (i) determination of the time‐averaged concentration of plasma‐generated RONS in the gas phase.

The configuration of the cold plasma soft jet used in this investigation is shown in Figure 1a. Air at room temperature, a high‐voltage electrode, a ground electrode, a high‐voltage power source and dielectrics were employed to create the plasma. A voltage controller was used to control the primary voltage, and the feed air gas flow rate was maintained at a steady 2 litres per minute (lpm). A high‐voltage electrode was made of stainless steel, measuring 1.20 mm by 0.27 mm, served as the inner electrode. Stainless steel was also used to make the ground electrode, which had dimensions of 6.00 mm in length, 0.27 mm in thickness and a 0.70 mm centrally drilled hole for plasma formation. A 6 mm space was kept between the tip of the plasma jet and the media, while a 2 mm discharge gap was maintained between the inner and outer electrodes. Peak voltage and current were measured using a digital oscilloscope (WaveSurfer 434, LeCroy, New York, NY, USA) equipped with a high‐voltage probe (P6015A, Tektronix, Beaverton, OR, USA) and a current probe (CP030, LeCroy). Using a fibre optic spectrometer (HR4000, Ocean Optics, Orlando, FL, USA), optical emission spectra (OES) and the study of RONS produced by the cold plasma soft jet were carried out. The electron temperature (kT_e_), electron density (N_e_) and the densities of nitrogen meta‐stable states (N_A_, N_B_ and N_C_) were measured using a modified Nitrogen collisional radiative model, as extensively elucidated in our previous study (Acharya, Lamichhane, et al., 2023). Additionally, the rotational temperature (Tg) and vibrational temperature (Tv) of the plasma were calculated using a Boltzmann plot, specifically employing the nitrogen second positive system, as previously described in our earlier work (Lamichhane et al., 2023). Gas‐phase Fourier transform infrared spectroscopy (FTIR) was used along with OPUS GA software and the automated FTIR gas analyser MATRIX‐MG5 to analyse the plasma in real‐time. For continuous real‐time measurement of plasma composition, the gas cell was coupled to the gas output of the cold plasma soft jet system and kept at a constant temperature of 25°C during the investigation.

Moreover, the quantification of H_2_O_2_ and NO_2_^−^ in plasma‐treated water was conducted utilizing a peroxide assay kit (DIOX‐250) and a nitric oxide assay kit (D2NO‐100), respectively. Detailed methodologies for these measurements are outlined in our recently published study (Borkar et al., 2024).

### 2.2 Cell lines and treatments

A253 cell line was grown in McCoy's 5A (Gibco, Waltham, MA, USA), HaCaT cell line in DMEM (Dulbecco's Modified Eagle Medium, Gibco), while HSC3 and HSC4 cell lines in RPMI (Roswell Park Memorial Institute Medium; Gibco). All the culture media were supplemented with 10% heat‐inactivated foetal bovine serum (FBS; Gibco), and 100 units/mL penicillin, and 100 μg/mL streptomycin (Sigma‐Aldrich, Merck, Milan, Italy). HaCaT was purchased from CliniSciences (#T0020001; France), while A253 cell line from American Type Culture Collection (ATCC #HTB‐41, Rockville, MD, USA); HSC3 and HSC4 cell lines were a kind gift from Prof. Lorenzo Lo Muzio (University of Foggia, Italy) and have been authenticated by short tandem repeat (STR) DNA genotyping (Figure S1a,b). All cell lines were cultivated at most for 1 month and maintained at 37°C in humified air with 5% CO_2_.

For CAP indirect treatments, PAM were obtained after exposing serum‐free media to CAP. The working distance between the capillary of the plasma device and the liquid medium surface was fixed at 6 mm. Two different culture media volumes—2 and 5 mL—were activated for 5, 10 and 20 min of exposure to CAP. Immediately after the activation, heat‐inactivated FBS was added to each PAM to a final concentration of 10%, according to the growth conditions of cells treated. Treatments were performed by incubating cells with the distinct PAM. Doxorubicin (#D5220, CliniSciences, Guidonia Montecelio, Roma, Italy) treatment, considered as the positive control, were carried in complete medium at a final concentration of 1 μM.

### 2.3 Cell proliferation assay

Cell viability was determined by MTS [3‐(4,5‐dimethylthiazol‐2‐yl)‐5‐(3‐carboxymethoxyphenyl)‐2‐(4‐sulfophenyl)‐2H‐tetrazolium] assay (Promega, Madison WI, USA; # G3581). Briefly, the day before PAM and doxorubicin (Doxo) treatments, cells were seeded and grown under standard culture conditions in 96‐well plates. HSC3, HSC4 and A253 cells were seeded at a density of 5 × 10^3^ cells per well, whereas HaCaT cells at a density of 6 × 10^3^ cells per well. After 24 h, the medium in each well was replaced by 0.1 mL of PAM or 0.1 mL of 1 μM Doxo. After 24, 48 and 72 h, the MTS solution was directly added to each well at a final concentration of 10% (v/v) and incubated for 1 h at 37°C. Cell viability was evaluated by measuring the colorimetric absorbance at 490 nm using a multi‐plate reader (Tecan trading AG, Switzerland). Cell viability was expressed as the percentage of inhibition of cell proliferation over the cell inhibition of control cells at 24, 48 and 72 h. Three independent experiments were performed, each carried out in technical triplicate.

### 2.4 pH measurement

Two different volumes (2 and 5 mL) of the three media used for the cell culture, namely DMEM for HaCaT, McCoy's 5A for A253 and RPMI for HSC3 and HSC4, were activated by exposure to CAP treatment for 5, 10 and 20 min (working distance 6 mm). The pH was measured after the addition of 5% FBS for PAM‐DMEM and 10% FBS to PAM‐McCoy's 5A and to PAM‐RPMI, at 0, 24, 48 and 72 h by Five Easy pH meter F20 (METTLER TOLEDO®, UK). The PAM were kept at 37°C and 5% CO_2_ for the duration of the experiment.

### 2.5 Ion chromatography

#### 2.5.1 Chemicals

Fluoride, chlorite, bromate, chloride, nitrite, bromide, chlorate, nitrate, phosphate and sulphate were purchased from Sigma Aldrich (Merck, Milan, Italy) and used without further purification. Sodium carbonate (analytical grade) used for the eluent phase was acquired by Sigma Aldrich. Double‐distilled water was obtained using a Millipore Milli‐Q Plus water treatment system (Millipore Bedford Corp., Bedford, MA, USA).

#### 2.5.2 Ion chromatography conditions

Ion chromatography analyses were performed on a Dionex ICS 1600 equipped with a thermostated AS autosampler and a DS6 heated conductivity cell detector. Chromeleon Software (Thermo Fisher Scientific) was used for data acquisition and elaboration. The autosampler was thermostated at 25°C ± 1°C. A Dionex IonPac AS9‐HC column (4 × 250 mm) was thermostated at 30 ± 1°C in a column oven and was used for the separation. A Dionex AERS500 carbonate (4 mm) electrolytically regenerated suppressor was used before the conductivity cell and an electrical current of 58 mA was used during the analyses. The acquisition was performed using a DS6 heated conductivity cell detector thermostated at 30°C ± 1°C with autozero enabled. The injection volume was 25 μL. The mobile phase was directly *on‐line* degassed, and isocratic elution was performed using the mobile phase sodium carbonate (9 mM) at the flow rate of 1 mL/min. All the prepared sample solutions were centrifuged, filtered on PTFE filter (0.45 μm) and the supernatant was injected into the system. The quantitative analyses were achieved by comparison of the analyte peak area (IS*minutes) versus an external calibration performed on analytes mix solution at different concentration levels obtained by dilution in the mobile phase (sodium carbonate, 9 mM). A robust baseline separation was achieved in 30 min. Under these conditions, the retention time of analytes were 3.882, 5.336, 6.061, 6.873, 8.741, 11.418, 12.569, 13.626, 18.466 and 21.457 min for fluoride, chlorite, bromate, chloride, nitrite, bromide, chlorate, nitrate, phosphate and sulphate, respectively.

#### 2.5.3 Preparation of standard solutions

The stock solutions of 10 anion standards were made in mobile phase (sodium carbonate, 9 mM) and the working solutions used for the validation procedure were made by dilution of stock solution in volumetric flasks with the mobile phase. Then the standards were injected into the system.

#### 2.5.4 Sample preparation

Two and 5 mL of the three media used for the cell culture, namely DMEM for HaCaT, McCoy's 5A for A253 and RPMI for HSC3 and HSC4, were exposed to CAP treatment for 5, 10 and 20 min (working distance 6 mm).

Then a kinetic evaluation was performed at 0, 24, 48 and 72 h. The samples were kept at 37°C for the entire experimental time. The PKSolver application for Excel and the IV infusion and non‐compartmental model (dose equal to 0.18 J/s) were used and applied on the concentration versus time curves obtained after the analysis of the different media.

### 2.6 Flow cytometry apoptosis detection

To assess apoptosis, a FITC Annexin‐V apoptosis detection kit (BD Pharmingen, San Diego, CA, USA) was used following the manufacturer's instructions. The day before PAM treatment, cells were seeded and grown under standard culture conditions in six‐well plates. HaCaT cells were seeded at a density of 1.7× 10^5^ cells per well whereas HSC3, HSC4 and A253 cells at a density of 2 × 10^5^ cells per well. After 24 h, 2 mL per well of each PAM considered were added to cells, which were further incubated. After 24 h, 10^5^ cells were gently re‐suspended in 100 μL of 1X binding buffer and incubated for 15 min at room temperature in the dark with 5 μL of Annexin‐V‐FITC and 5 μL of Propidium Iodide (PI). After the addition of 200 μL of 1X binding buffer, samples were analysed with a Cytoflex flow cytometer with the FL1 and FL3 detector in a log mode, using the Cytoexpert analysis software (both from Beckmann Coulter, Milan, Italy). For each sample, at least 5000 events were collected. Viable cells are Annexin‐V^neg^/PI^neg^, early apoptotic cells are Annexin‐V^pos^/PI^neg^, late apoptotic cells are Annexin‐V^pos^/PI^neg^ and dead cells are Annexin‐V^neg^/PI^pos^.

### 2.7 Flow cytometry cell cycle analysis

Briefly, the day before PAM treatments, cells were seeded and grown under standard culture conditions in six‐well plates. HSC4 cells were seeded at a density of 2 × 10^5^ cells per well, whereas HaCaT cells at a density of 1.7× 10^5^ cells per well. After 24 h, 2 mL per well of each PAM considered were added to cells and further incubated. After 24 h, 3 × 10^5^ cells per experimental condition were harvested, fixed in 70% cold ethanol, and kept at 4°C overnight. The cells were centrifuged and re‐suspended at 5 μg/mL PI and 100 μg/mL RNAse. Cell cycle profiles (at least 5000 events) were analysed by a CytoFLEX flow cytometer with the FL3 detector in a linear mode using the CytoExpert analysis software (both from Beckmann Coulter). Data were analysed with ModFit software (Verity Software House, ME, USA).

### 2.8 Flow cytometry ROS analysis

Reactive Oxygen Species production was determined by monitoring by flow cytometry the increase of green fluorescence after labeling the cells (5 × 10^5^) with 5 μM of 5‐(and‐6)‐chloromethyl‐2′,7′‐dichlorodihydrofluorescein diacetate, acetyl ester (CM‐H2DCFDA, cat. C6827, Molecular Probes, Invitrogen, Life‐Sciences‐Division, Milano, Italy) for 1 h at 37°C, as already described (Giampietro et al., 2019). When ROS are produced, an increase in green fluorescence is detected by a Cytoflex cytometer with an FL1 detector in a log mode using the Cytoexpert software (Beckmann Coulter, FL, USA). The median fluorescence intensity (MFI) ratio was obtained by histogram statistics and provided to quantify ROS production. Dead cells were excluded from analysis by Propidium Iodide (PI) staining (5 μg/mL) (cat. P4864, Sigma‐Aldrich). At least 5000 events for each sample were acquired.

### 2.9 Statistical analysis

The data are reported as the representative values of three independent experiments. Data are expressed as means ± SD and analysed by two‐way ANOVA statistical test. A value of at least *p* < 0.05 was considered to indicate a statistically significant difference.

## 3 RESULTS

### 3.1 Electrical, optical, thermal properties of plasma

Figure 1a illustrates the soft jet plasma setup, while Figure 1b displays discharge waveforms in air, showing typical current and voltage patterns. Peak voltage was measured at 1.6 kV, while peak current is 1.5 A. Voltage regulation involves a 12% duty ratio with 31 milliseconds of on‐time. The plasma operates at a frequency of 83 kHz, delivering electrical energy at 0.17 J/s and a power of 1.7 W (Figure 1c). Duty ratio, energy generation, power dissipation for the plasma were calculated using the following Equations ((1), (2), (3)) (Acharya et al., 2022; Pradeep Lamichhane et al., 2022).

(1) $$\text{Duty}\;\text{ratio}=\frac{\mathrm{On}\;\text{time}}{\left(\mathrm{On}\;\text{time}+\mathrm{Off}\;\text{time}\right)}\times 100\%$$

(2) $$\text{Energy}\;\left(E\right)=Q\times V=\int_{T1}^{T2}V\left(t\right)I\left(t\right)\mathrm{dt}\;\left[J\right]$$

(3) $$\text{Power}\;\left(P\right)=\text{Duty}\;\text{ratio}\times \frac{1}{T}\;\int_{0}^{T}V\left(t\right)I\left(t\right)\mathrm{dt}\;\left[W\right]$$

where, Q = charge, V = peak voltage, I = peak current, T = plasma discharge time.

Figure 1d provides the optical emission spectra (OES) of the soft jet plasma used in this study over a wavelength range from 200 to 1000 nm. In the 200 to 280 nm range, the NOX spectrum was observed. The emission of OH radicals at 309 nm was seen. The emission attributed to the nitrogen second positive (N2 SPS) was evident in the 311–380 nm region (Acharya, Jang, et al., 2023). Additionally, emissions from the first negative system (FNS) of nitrogen (N_2_) were observed between 390 and 410 nm. Further, within the 550–700 nm range, emissions from the first positive system (FPS) were observed. Notably, atomic nitrogen (N) emitted distinct light at 780 nm, while atomic oxygen (O) emission was observed spanning the range from 777 to 845 nm. The electron temperature (kTe) was quantified at 1.2 eV, while the electron density (Ne) was ascertained to be 2.82 × 1014 cm^−3^. Correspondingly, the densities of nitrogen meta‐stable states (NA, NB and NC) were identified at 2.82 × 1015 cm^−3^, 2.10 × 1015 cm^−3^ and 1.35 × 1014 cm^−3^, respectively. Additionally, the vibrational temperature (Tv) and rotational temperature (Tg) of the cold soft jet plasma were computed at 0.7 eV and 742 K, respectively. These elevated electrons, vibrational and gas temperatures, in comparison to other plasma devices, establish the soft jet plasma as a prolific generator of an abundant quantity of reactive species, with a notable emphasis on NO production (Acharya, Jang, et al., 2023; Dhakal et al., 2023). Furthermore, the determination of electron densities in nitrogen metastable states A, B and C is pivotal in understanding the generation of RONS and can significantly influence plasma reactivity. In cold plasma, the higher these densities, the greater the formation of RONS (Acharya, Lamichhane, et al., 2023).

The findings of an examination using FTIR spectroscopy to determine the RONS in the gas phase of the plasma are shown in Figure 1e. The presence of several gases RONS by the plasma was shown by the FTIR spectra, which ranged from 3000 to 1000 cm^−1^. These gases included NO (1700–2000 cm^−1^), NO_2_ (1540–1660 cm^−1^ and 2840–2940 cm^−1^) and N_2_O (1250–1350 cm^−1^). Figure 1f–h demonstrated how the concentrations of plasma‐generated NO, NO_2_ and N_2_O changed before and after plasma treatment during a 12min timeframe. Notably, during the initial 0–2 min, no reactive species were detected, indicating their absence before the plasma was generated. However, after initiating the plasma synthesis from 2 to 12 min, distinct reactive species, especially NO, NO_2_ and N_2_O, became evident, signifying their generation within this specific period. Notably, NO displayed a predominant concentration, averaging approximately 130.1 ppm over the 10 min plasma treatment. In contrast, NO_2_ and N_2_O exhibited lower average concentrations of about 45.0 and 2.9 ppm, respectively, during the same 10 min plasma exposure (Figure 1I). Furthermore, this study investigated a correlation between exposure time and RONS generation in the liquid phase, with concentrations peaking after 20 min of exposure in a dose‐dependent manner. Specifically, in a 5 mL volume of deionized water, concentrations of H_2_O_2_ following 2, 10 and 20 min of exposure were determined to be 6, 25 and 40 μM, respectively. Similarly, concentrations of NO_2_^−^ following 2, 10 and 20 min of exposure were measured at 104, 1010 and 1630 μM, respectively.

### 3.2 PAM exhibited selective and cytotoxic effects on HNC cells

**FIGURE 2:**
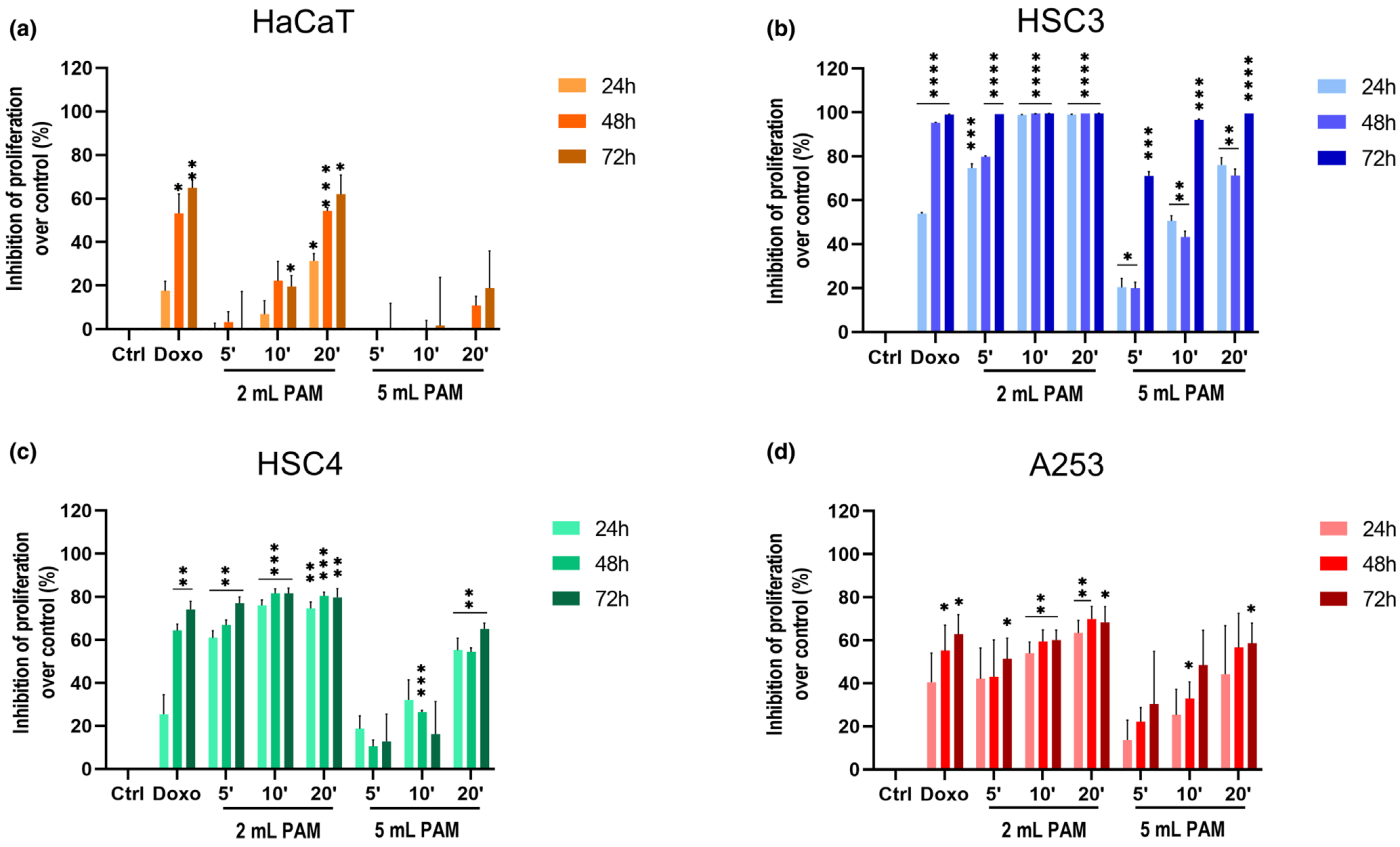
Inhibitory effects of PAM on (a) normal human epidermal keratinocytes (HaCaT) and on three HNC cell lines: (b) HSC3, (c) HSC4 and (d) A253 proliferation rates. Histograms represent the inhibition of proliferation over the cell inhibition of control cells at 24, 48 and 72 h (expressed as 0% of inhibition). Doxorubicin (Doxo) 1 μM treatment was used as positive control. *p < 0.05 versus Ctrl, **p < 0.01 versus Ctrl, ***p < 0.001 versus Ctrl and ^****^ *p* < 0.0001 versus Ctrl.

In order to evaluate the selective cytotoxic effect of PAM toward cancer cells, MTS assays were performed on three HNC cell lines (HSC3, HSC4 and A253), as well as on normal HaCaT treated or not with different PAM, which were obtained by exposing different volumes—2 or 5 mL—of cell culture media to CAP for 5, 10 or 20 min. All the cell lines considered were treated with the different PAM, and inhibition of cell proliferation was evaluated at distinct time points (24, 48 and 72 h). As a positive control, cells were also treated with 1 μM Doxo (Figure 2a–d).

Doxo treatment showed a significant reduction of cell proliferation for all cells, as expected, including HaCaT, at either 48 or 72 h, whereas PAM exhibited a cytotoxic effect on all HNC cell lines tested (Figure 2a–d). In particular, all the 2 mL conditions showed a significant reduction in cell proliferation in all the tumoral cell lines tested, with an 100% of inhibition in almost all the experimental points for HSC3 and about 70% of inhibition in HSC4 cells, when compared to each control. As for A253 cells, the 2 mL PAM samples showed a less marked inhibitory effect than in the two other tumoral cell lines, but the 10 and 20 min 2 mL PAM still exerted a significant inhibitory effect of about 50%. Interestingly, the primary cells HaCaT were slightly affected by the 2 mL PAM, even though the 20 min 2 mL PAM induced greater rates of cytotoxicity than the other exposure times.

On the other hand, the treatment with the 5 mL PAM induced a milder effect on cellular proliferation compared to that exerted by the exposure to 2 mL PAM (Table S1). In particular, for both HSC3 and HSC4 cell lines, the inhibition exerted by the 5 mL PAM was remarkably lower than that observed in 2 mL treated samples, either at 24 or 48 h. In addition, a time‐dependent correlation could be observed in HNC cells, while HaCaT cells were unaffected by 5 mL PAM.

### 3.3 pH value changes were not related to cell proliferation inhibition

**FIGURE 3:**
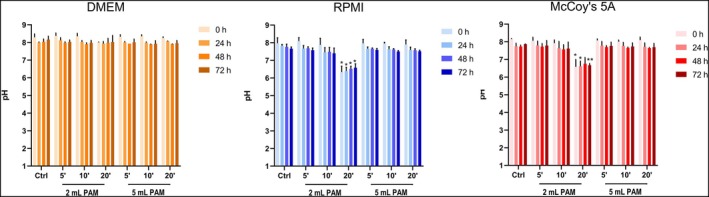
pH values at different time points (24, 48 and 72 h) after exposing DMEM, RPMI and Mc Coy's 5A media to CAP treatment. *p < 0.05 and **p < 0.01 versus Ctrl same experimental time.

To assess whether the chemical characteristic of the PAM could be related to their biological effects, the pH of the three culture media used for cell culture, namely DMEM for HaCaT, McCoy's 5A for A253 and RPMI for HSC3 and HSC4, was measured at the same experimental points of the MTS assay. The pH of DMEM‐PAM does not show any changes, whereas a significant decrease in the 2 mL 20 min sample was found either in RPMI‐ or McCoy's 5A‐PAM in all the experimental times (Figure 3).

### 3.4 PAM chemical composition by ion chromatography

**FIGURE 4:**
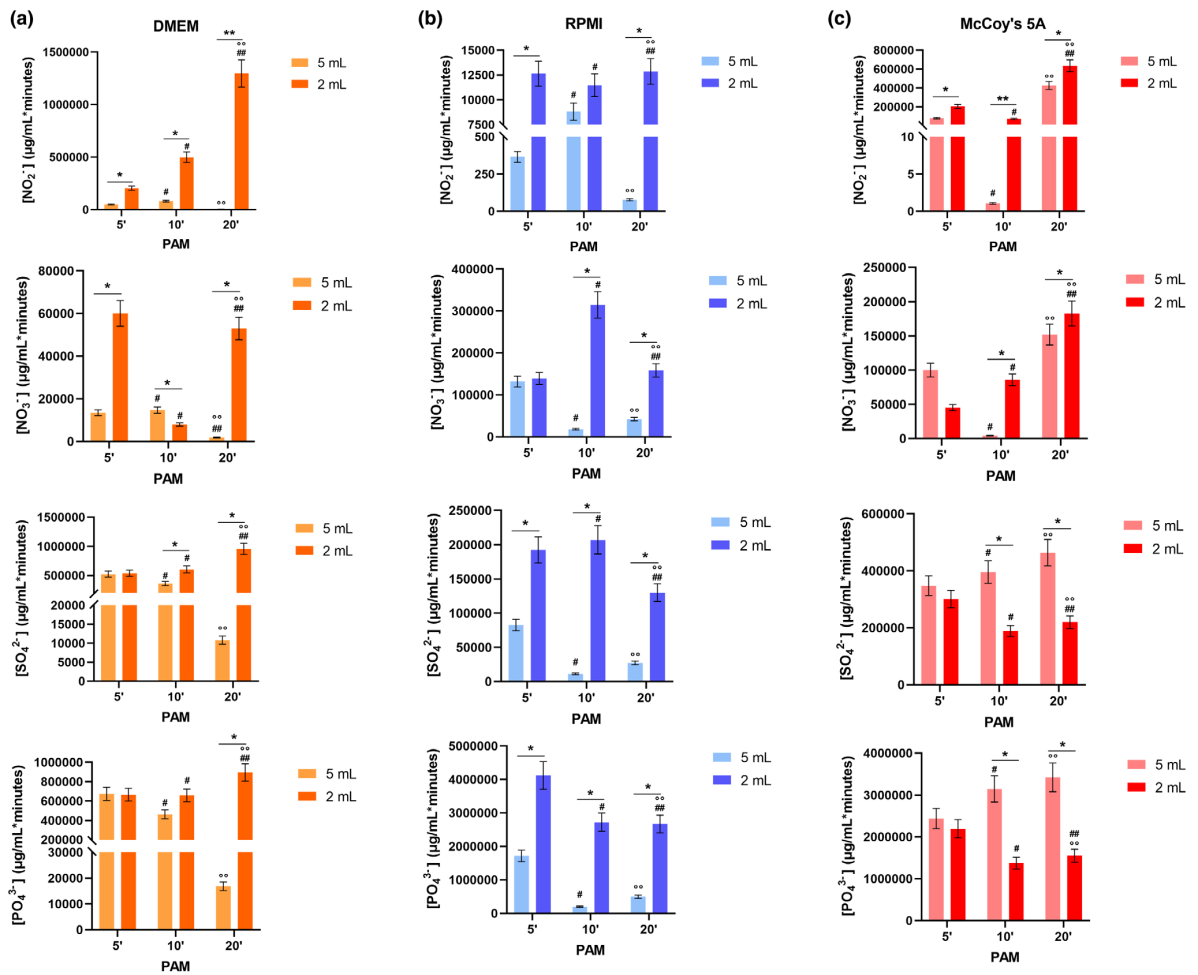
Area under curve (AUC) for the considered anions (NO_2_^−^, NO_3_^−^, SO_4_^2−^, PO_4_^2−^) submitted to PKSolver data elaboration in DMEM, RPMI and McCoy's 5A media after exposure to CAP treatment. Statistical significance: *p < 0.05 2 mL versus 5 mL; **p < 0.01 2 mL versus 5 mL; ^#^ *p* < 0.01 10′ versus 5′; ^##^ *p* < 0.01 20′ versus 5′; ^°°^ *p* < 0.01 20′ versus 10′.

Ion chromatography was applied to quantify the individual ionic species produced in all the PAM used for the biological experiments, namely DMEM–PAM for HaCaT, McCoy's 5A‐PAM for A253 and RPMI‐PAM for HSC3 and HSC4. This method has been validated according to international guidelines (Bioanalytical Method Validation Guidance for Industry FDA, 2018; European Medicines Agency, 2019; ICH Official web site: ICH, 2023.) in order to guarantee the quality of the analytical data (Table S2). In the effort to standardize the analysis of PAM chemistry, a kinetic evaluation was performed at 0, 24, 48 and 72 h; the specific results for nitrite (NO_2_^−^), nitrate (NO_3_^−^), sulphate (SO_4_^2−^) and phosphate (PO_4_^2−^) content are reported in Figure 4 and Tables S3–S6. A volume‐dependent increase of the analysed anions concentration was observed in all PAM, except for sulphates and phosphates in McCoy's 5APAM. In a few cases the model was not able to fit the experimental values, therefore the parameters were not evaluated, and the values are missing in the tables.

### 3.5 PAM induce apoptosis occurrence in HNC cell lines

**FIGURE 5:**
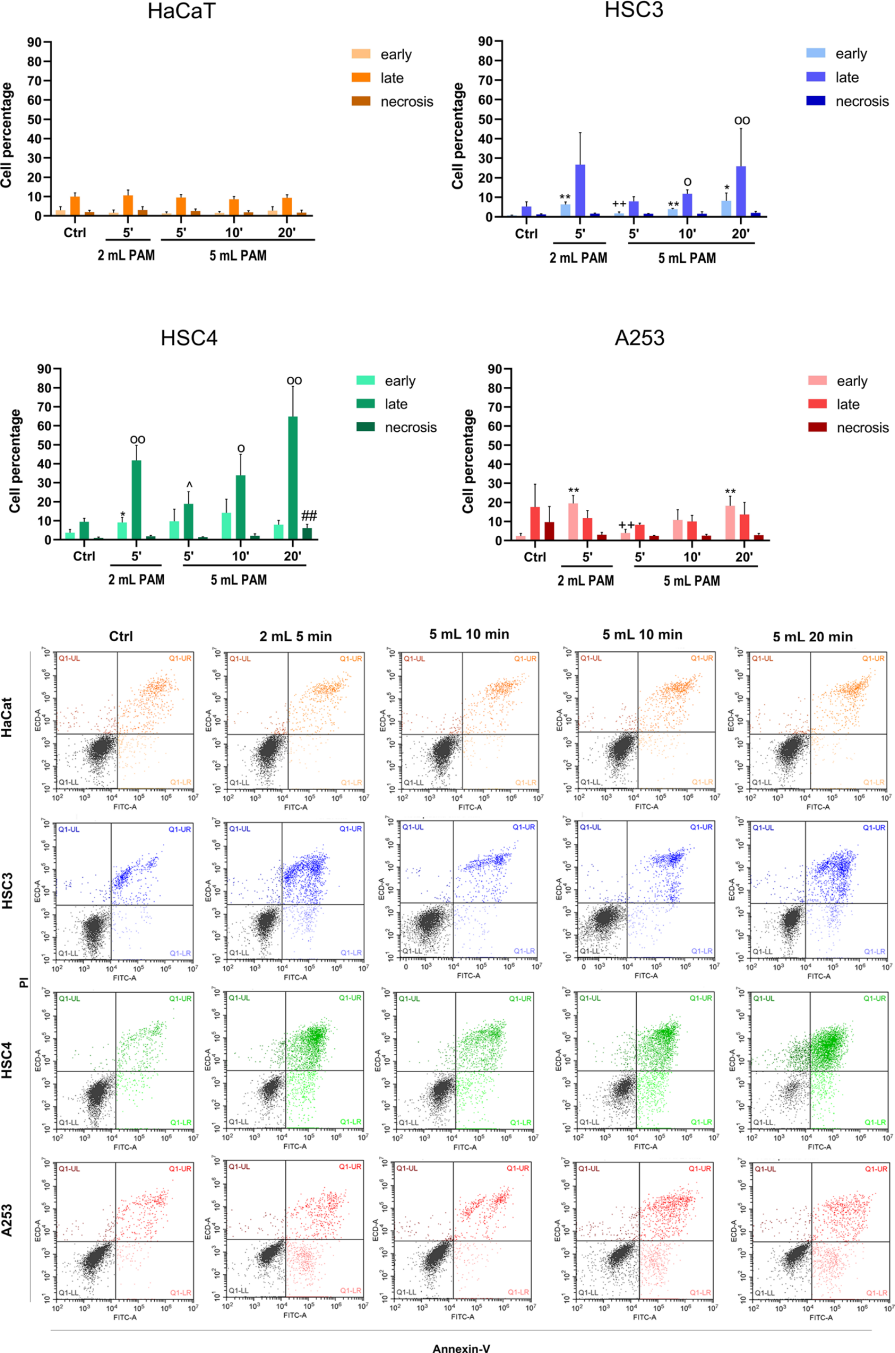
Effects of PAM in the induction of apoptosis of normal cells (HaCaT) and three HNC cell lines (HSC4, HSC3 and A 253). Histograms represent the percentage of early and late apoptosis and necrosis at 24 h. Representative cytometer dot plots for each cell type are shown (bottom). *p < 0.05 versus Ctrl (early apoptosis), **p < 0.01 versus Ctrl (early apoptosis), ^o^ *p* < 0.05 versus Ctrl (late apoptosis), ^oo^ *p* < 0.01 versus Ctrl (late apoptosis), ^#^ *p* < 0.05 versus Ctrl (necrosis), ^##^ *p* < 0.01 versus Ctrl (necrosis), ^^^ *p* < 0.05 versus 5 min 2 mL (late apoptosis), ^++^ *p* < 0.01 versus 5 min 2 mL (early apoptosis) refer to statistical significance analysed with two‐way ANOVA.

Since the oxidative stress at the basis of the antitumoral effects of PAM can lead to apoptosis, its occurrence was investigated in the same four different cell types. Based upon the previous findings, apoptosis induction was analysed by flow cytometry after 24 h of treatment with the distinct PAM. To evaluate apoptosis, only 5, 10, 20 min 5 mL volumes and 5 min 2 mL volume were considered, since the 10 and 20 min 2 mL PAM showed a too high percentage of cell proliferation inhibition (Figure 5).

HSC4 cells showed the highest sensitivity to PAM‐induced apoptosis among all the analysed tumoral cell lines, while A253 was the least sensitive one. In HSC4, the highest percentage of late apoptosis was observed after 20 min 5 mL PAM and 5 min 2 mL PAM treatments, while in HSC3 and A253 cells apoptosis was induced in a comparable manner by the 5 min 2 mL and 20 min 5 mL. On the other hand, the apoptotic effects exerted by 5, 10 and 20 min 5 mL PAM showed an increasing time‐dependent trend, especially in HSC4 cell line, although no significance was found. Interestingly, 5 min 2 mL PAM was found able to induce higher apoptosis percentages than 5 mL exposed to CAP for the same time span, suggesting that the effects of PAM depended on the volume of the medium treated by CAP.

In line with cell proliferation results, HaCaT cells showed a very low percentage of apoptosis in all the experimental points, supporting that our approach has a selective effect on cancer cells.

In summary, the exposure to PAM induces apoptosis occurrence, preferentially in cancerous cell lines. Five minutes 2 mL and the 20 min 5 mL PAM emerged among the other treatment conditions as the most promising in terms of cytotoxic effects.

### 3.6 PAM induce selective cell cycle changes

**FIGURE 6:**
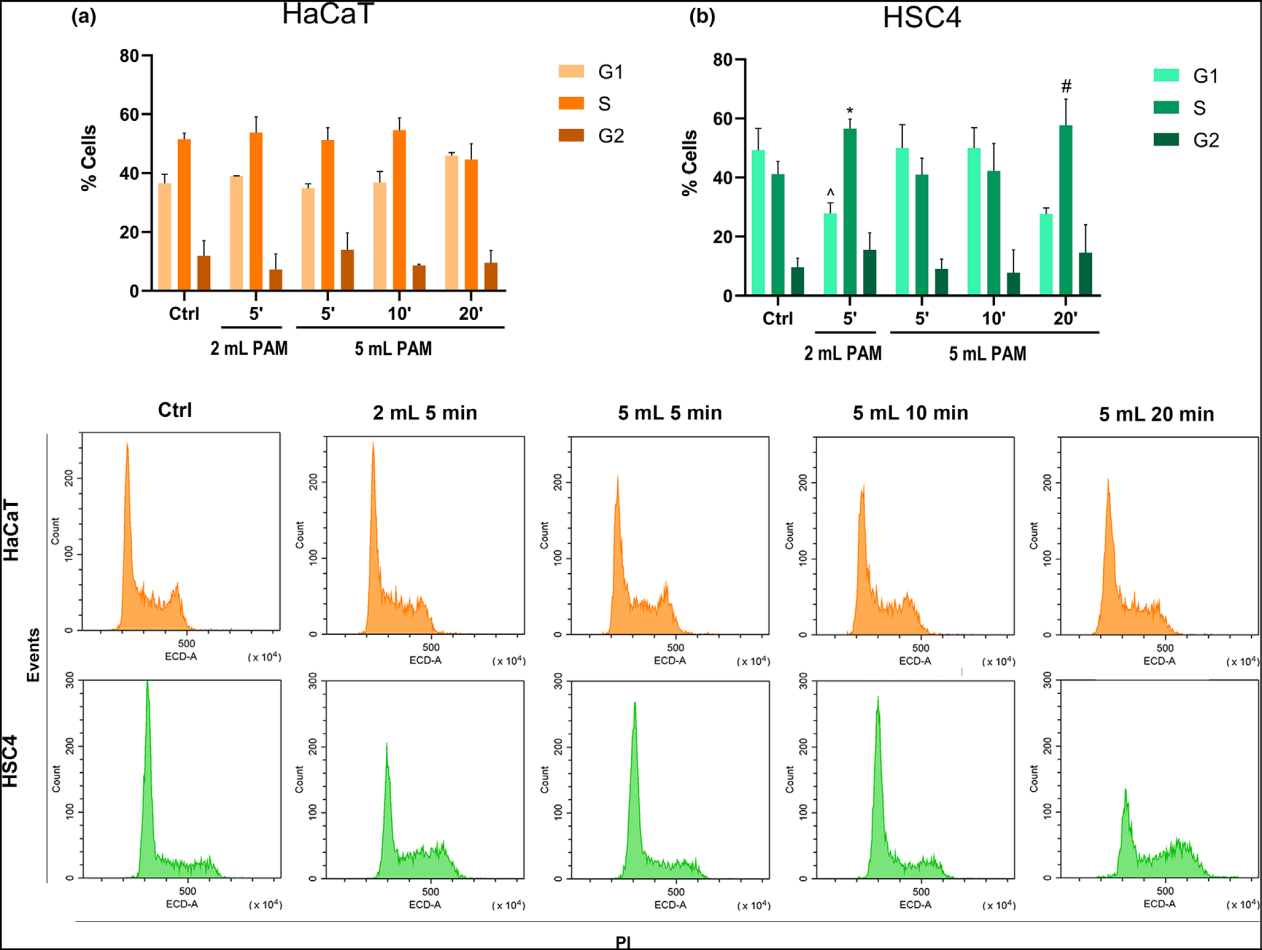
Effects of PAM on cell cycle of (a) normal cells (HaCaT) and (b) tumoral HSC4 cell lines. Histograms (above) represent the percentage of G_1_, S and G_2_/M phases at 24 h. Representative cytometer histograms for each cell type are shown (bottom). ^^^ *p* < 0.05 versus 5 mL 5′ G_2_; ^#^ *p* < 0.5 versus 5 mL 10′ G_2_; *p < 0.05 versus ctrl S;°*p* < 0.05 versus Ctrl G_1_ refer to statistical significance analysed with two‐way ANOVA.

HSC3 and A253 cell lines were excluded by the cell cycle analysis because the first was too sensitive to plasma treatment, reaching peaks of 100% inhibition of their proliferation, and the latter because was not enough sensitive to plasma treatment. In line with previous results, cell cycle of HaCaT cells was not affected by the exposure to CAP, while the most extreme experimental conditions, namely 5 min 2 mL and 20 min 5 mL, induced a decrease of the percentages of HSC4 cells in G_1_ phase counteracted by an increase of HSC4 cells in S phase (Figure 6).

### 3.7 PAM induce intracellular ROS production

**FIGURE 7:**
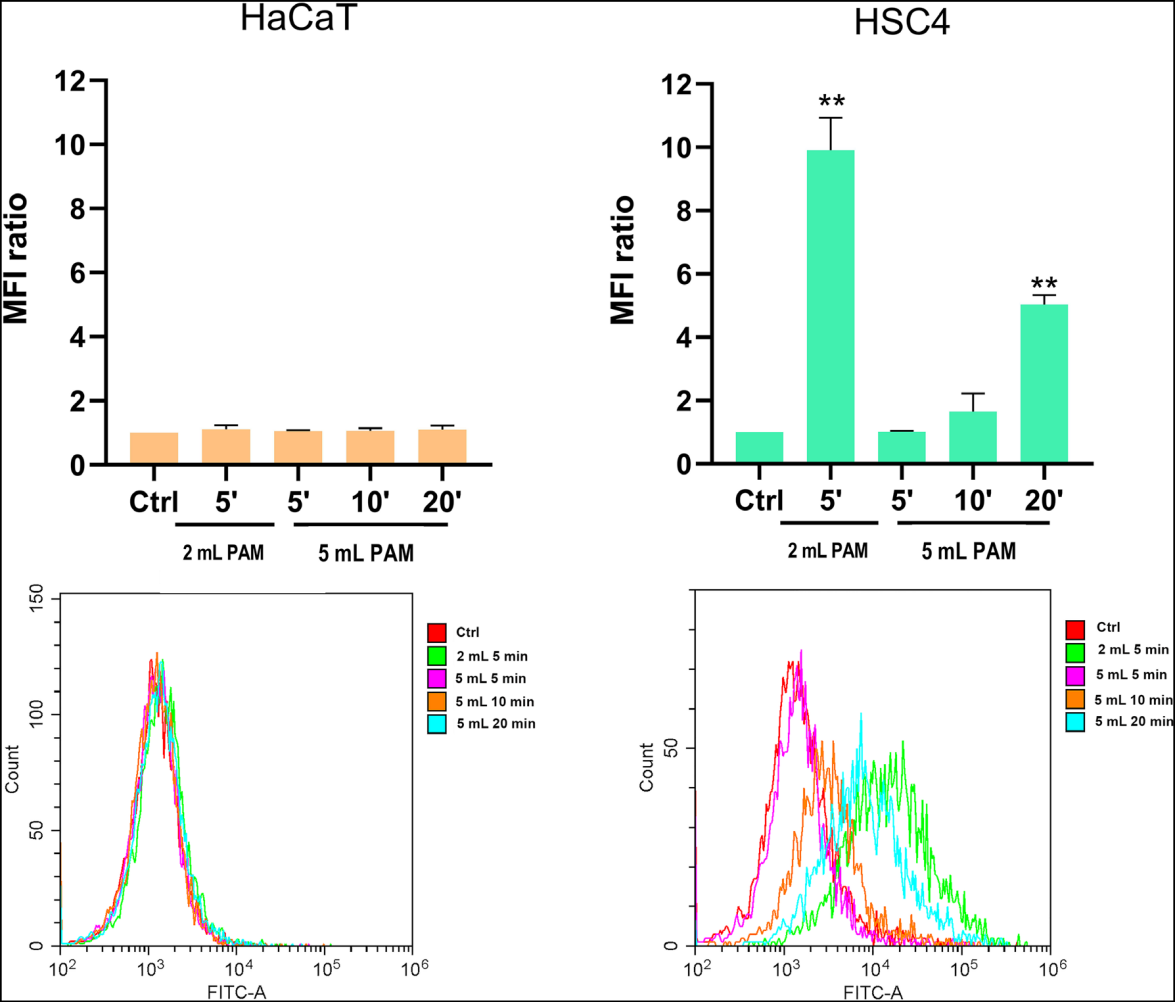
Effects of PAM on intracellular ROS production of normal cells (HaCaT) and tumoral HSC4 cell line. Histograms (above) represent the median fluorescence intensity (MFI) ratio over control at 24 h. A representative overlay plot for each cell type is shown (bottom). **p < 0.01 versus Ctrl refers to statistical significance analysed with two‐way ANOVA.

Since CAP antitumoral effects are mediated by ROS, their intracellular production was measured both in HaCaT and HSC4. As for the former, in all the experimental conditions the ROS levels show no significant changes, whereas the most effective experimental conditions—5 mL 20 min and 2 mL 5 min—were able to increase the ROS intracellular concentration in the tumoral cell line HSC4 from four to almost eight‐fold when compared to the untreated cells. A slight increase was found also in 5 mL 10 min, suggesting a time‐dependent effect in the 5 mL treated samples (Figure 7).

## 4 DISCUSSION

CAP application in oncology is currently a field of interest (Limanowski et al., 2022) and a great effort is aimed at analysing CAP efficacy against the most insidious cancers (Murthy et al., 2022; Privat‐Maldonado et al., 2022). Indeed, plasma treatment was not only found effective in in vitro models, but it has showed promising effects also in vivo models of cholangiocarcinoma, a rare and very aggressive cancer emerging from the biliary tree (Vaquero et al., 2020), and other tumors (Dezhpour et al., 2023; He et al., 2023), because it can exert its beneficial activity by modulating the tumor microenvironment (Dai & Zhu, 2023). Among the others, HNC represent a challenge due to their heterogeneity and poor response to standard anti‐cancer treatments (Perrotti et al., 2022). Three different cell lines, namely HSC3, HSC4 and A253, were chosen for our study because their response to CAP treatment has not been investigated yet. Even though their proliferation was significantly inhibited, some differences can be found among the three types of cells. A253, which is a cellular model of submandibular gland squamous cell carcinoma, appeared to be more resistant to the PAM treatment, whereas the HSC3, a cell line derived from an oral cavity squamous cell carcinoma, showed a very high inhibition of cell proliferation, even in comparison with the tongue squamous cell carcinoma‐derived HSC4 cell line. This discrepancy can be easily attributed to the different cell types, which affect cell response, as demonstrated in other experimental setups. Indeed, HSC3 and HSC4 cells show a different metabolism which is a well‐known mediator of immunological reactions and performs a variety of functions via autocrine and paracrine mechanisms (Kon et al., 2022) and a different expression of EPSTI1, whose overexpression is considered to drive the malignant phenotype (Fan et al., 2022). On the other hand, HaCaT seem only slightly affected by the plasma exposure, confirming the selectivity of our approach, that is, targeting cancer cells with milder effects on primary cells, as already reported in the literature for other tumor models (Hamouda et al., 2021). Indeed, plasma selectivity is based on three major differences between normal and cancer cell lines: (1) cancer cells have high intracellular residual ROS, as compared to normal cells; (2) cancer cells have weak antioxidant activity as compared to normal cells; (3) cancer cells have more pores and receptors for ROS delivery than normal cells. Therefore, plasma delivered RONS will be a burden on cancer cells and induce the cascade of cell death (Keidar, 2018; Yan et al., 2017).

One limitation of our study is the fact that HaCat is a spontaneously transformed aneuploid immortal keratinocyte cell line derived from adult human skin, hence not the completely appropriate healthy counterpart of the tumoral cell lines. One significant dissimilarity between these cell types is the response to injury: oral mucosal wounds heal faster and with less inflammation than equivalent cutaneous wounds. However, in this study, the parameters evaluated are not related to regenerative ability rather than to the response to oxidative stress induced by plasma treatment, which is distinctly different in normal and cancer cells.

Furthermore, human oral keratinocytes (HOK), obtained from the lining mucosa of the human oral cavity, are described as having notable structural and functional parallels to dermal keratinocytes. Consequently, even though HaCat differential response to CAP treatment can be considered a valid representation of normal cell behaviour with respect to tumoral cells, further studies focused on the thorough investigation of finely tuned molecular mechanisms are worth considering to be conducted on primary human oral keratinocytes.

Besides the selectivity, an interesting time‐dependent effect was found, meaning that a longer exposure of the media to CAP action results in a higher inhibition of cancer cells proliferation. Such results are in line with previous studies on a few cancer models (Hattori et al., 2015; Saadati et al., 2018; Utsumi et al., 2013). In addition, when 2 mL and 5 mL of the same culture medium were exposed to CAP for the same treatment time, the 2 mL exerted a stronger inhibitory effect on HSC3 and HSC4 than the 5 mL counterpart. Even though a confirmation of such volume‐dependent effect is hard to find in literature, due to the variability of experimental designs in this field of research, it was supported by our data on apoptosis occurrence. The programmed cell death via the intrinsic pathway has been reported among the cytotoxic effects exerted by CAP in some cancer cell lines (Hua et al., 2021; Jalili et al., 2016; Terefinko et al., 2021; Wang et al., 2022), but to the best of the authors' knowledge, this is the first time that it was demonstrated in the cancer cell lines selected for the present study. As for cell cycle analysis, the HSC4 cell line was chosen to be compared with the primary cells HaCaT, because of its sensitivity to plasma exposure, without the too strong effects registered for HSC3 cells. The impairment of G_1_ and S phases is somewhat discordant with other studies already present in literature (Hua et al., 2021; Volotskova et al., 2012), but Cheng and collaborators (Cheng et al., 2021) demonstrated that 8‐oxoG modification and degradation of histone mRNA during the early S phase of the cell cycle, is the primary cause of cell death in breast cancer treated with plasma. Once again, our findings support the selectivity of the plasma treatment, being the cancer cells, even though with the differences as discussed above, affected by the exposure to PAM, whereas keratynocyte viability, apoptosis occurrence, and cell cycle are unaffected. Considering that the lack of standardization is a major issue in Plasma Medicine, one bias in our study could be the use of different culture media. Therefore, a chemical characterization of the PAM generated by our experimental design was performed. First, although the increase in pH values observed at 0 h is in alignment with other studies (Sklias et al., 2021), a poor correlation to cell viability and apoptosis occurrence was evidenced. Consequently, an innovative approach by ion chromatography was used to determine the concentrations of chosen anions, namely nitrites, nitrates, sulphates and phosphates, in the different generated PAM. The advantage of this technique lies in the fact that it allows the quantitative analysis of multiple ionic species simultaneously in a single analysis with better accuracy than spectrophotometric and spectrofluorimetric techniques. Furthermore, compared to the latter, it does not present matrix effect problems which could lead to overestimation or underestimation of the ionic species considered. To our best knowledge, ion chromatography has been used for the first time to quantify anions in PAM and a pharmacokinetic approach has been adopted, which could enable the application of a “dose concept” to PAM. However, the methodology could be optimized by analysing PTL with a less complex chemical composition than culture media, to further refine the sensibility of the technique and widen the range of anions quantifiable in a single run.

Interestingly, in the present study ion chromatography showed a volume‐dependent increase of nitrites (NO_2_^−^) and nitrates (NO_3_^−^), which correlates with the volume‐dependent proliferation inhibition in cancer cells as already demonstrated in other cancer cell lines (Almeida‐Ferreira et al., 2022; Iuchi et al., 2018) where nitrites and nitrates, produced by NO via oxidation reaction, have an anti‐cancer activity (Bauer et al., 2019). In addition, our data showed that the increase in the production of nitrites correlated with the selective inhibitory effects on cancer cell proliferation. Indeed, in DMEM PAM the nitrites concentration was higher but the HaCaT proliferation was much less affected than tumoral cell lines. This finding is in agreement with Sardella et al. who found that nitrites showed selectivity in plasma‐treated Saos2 cancer cells versus endothelial cells (Sardella et al., 2021). As for the phosphate role in cytotoxicity, the literature is not clear, but significant higher ROS production was found when the phosphate buffer solution is activated by plasma, in comparison either to saline solution or to DMEM, suggesting that the phosphate anion can have a role in increasing the oxidative stress (Griseti et al., 2020; Wang et al., 2019). Our findings do not fully explain the inhibitory effects observed on A253 cells, but it is well known that the plasma anticancer activity is not completely related to RONS species production (Li et al., 2021).

## 5 CONCLUSIONS

The media chemical composition modified by CAP exposure influenced cell proliferation by modulating cell cycle and inducing apoptosis in cancer cells, without affecting normal cells.

As future perspectives, a deeper understanding of the correlation between chemical composition of the PAM generated under different experimental conditions and their biological effects is needed to translate this promising therapeutic approach to clinical practice.

Currently, CAP direct approach can either be used alone or in synergy with other conventional therapies for effective cancer treatment. As for chemotherapy, CAP showed the potential to produce synergistic effects both with gemcitabine (Masur et al., 2015) and cisplatin (Brunner et al., 2022). Furthermore, CAP was also used to reinstate the susceptivity of cancer chemoresistant cells (Köritzer et al., 2013) and to improve the efficacy of current immunotherapies in glioblastoma (Almeida et al., 2019). Interestingly, CAP treatment has been already successfully used as palliative treatment of patients affected by HNC, demonstrating a visible response to tumor surface (Almeida et al., 2019).

In the future, more research should be conducted on the indirect application of CAP in both animal and human models, because this modality could allow the delivery of the RONS species to the deeper layers of tumoural tissues.

## AUTHOR CONTRIBUTIONS

**Viviana di Giacomo:** Conceptualization; writing – original draft; visualization. **Marwa Balaha:** Methodology; data curation. **Morena Pinti:** Investigation. **Maria Carmela Di Marcantonio:** Investigation. **Ilaria Cela:** Investigation. **Tirtha Raj Acharya:** Methodology; formal analysis; writing – original draft. **Nagendra Kumar Kaushik:** Methodology; formal analysis; resources; funding acquisition. **Eun Ha Choi:** Methodology; resources; supervision; funding acquisition. **Gabriella Mincione:** Formal analysis. **Gianluca Sala:** Validation. **Miryam Perrucci:** Investigation. **Marcello Locatelli:** Formal analysis. **Vittoria Perrotti:** Conceptualization; writing – review and editing; project administration; funding acquisition.

## FUNDING INFORMATION

This research was funded by European Union ‐ NextGeneration EU ‐ MUR, Fondo Promozione e Sviluppo, DM 737/2021–Project title: COld atmospheric plasma therapy to target head and neck Tumours by A multImodaL approach; Acronym: Cocktail; CUP number: D75F21003210001. This study was also supported by the National Research Foundation (NRF) of Korea, funded by the Korean government 2021R1A6A1A03038785. This study was supported also by Fondazione AIRC to GS (IG 2021 id 25696).

## CONFLICT OF INTEREST STATEMENT

All authors have no conflicts of interest to disclose.

## Supporting information


Data S1.

## Data Availability

The data that support the findings of this study are available from the corresponding author upon reasonable request.
