# Supplementary material for: Cold atmospheric plasma activated media selectively affects human head and neck cancer cell lines
Source: Oral Dis. 2024 Sep 24;31(2):401–16. doi: 10.1111/odi.15120 (PMC11976116; doi:10.1111/odi.15120)
Supplement: Supplementary file 1 — Data S1. [file ODI-31-401-s001.docx]

Supplementary material

Cold atmospheric plasma activated media selectively affects human head and neck cancer cell lines

Viviana di Giacomo^1,2^; Marwa Balaha^3,4^; Morena Pinti^3^; Maria Carmela Di Marcantonio^5^; Ilaria Cela^5,6^; Tirtha Raj Acharya^7^; Nagendra Kumar Kaushik^7^; Eun Ha Choi^7^;; Gabriella Mincione^5^; Gianluca Sala^5,6^; Miryam Perrucci^1^; Marcello Locatelli^1^; Vittoria Perrotti^2,3^*

^1^ Department of Pharmacy, “G. d’Annunzio” University of Chieti-Pescara, Chieti, Via dei vestini, 31-66100 Chieti, Italy;

^2^ UdA-TechLab, Research Center, “G. d’Annunzio” University of Chieti-Pescara, Via dei vestini, 31-66100 Chieti, Italy; Chieti, Italy;

^3^ Department of Medical, Oral and Biotechnological Sciences, “G. d’Annunzio” University of Chieti-Pescara, Via dei vestini, 31-66100 Chieti, Italy;

^4^ Department of Pharmaceutical Chemistry, Faculty of Pharmacy, Kafrelsheikh University, 33516 Kafr El Sheikh, Egypt;

^5^ Department of Innovative Technologies in Medicine & Dentistry, University of Chieti-Pescara, Chieti, Italy;

^6^ Center for Advanced Studies and Technology (CAST), “G. d’Annunzio” University of Chieti-Pescara, Chieti, Italy;

^7^ Plasma Bioscience Research Center, Department of Electrical and Biological Physics, Kwangwoon University, Seoul 01897, South Korea;

^8^ Genetic Molecular Institute of CNR, Unit of Chieti, “G. d’Annunzio” University of Chieti-Pescara, Via dei vestini, 31-66100 Chieti, Italy.

**Figure S1:** STR cell line authentication of (A) HSC3 and (B) HSC4 cells

A


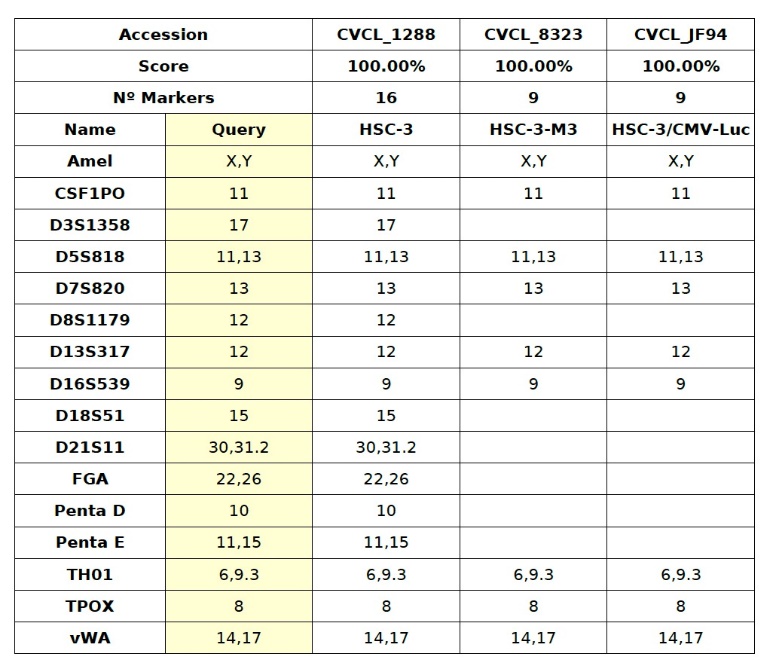


B


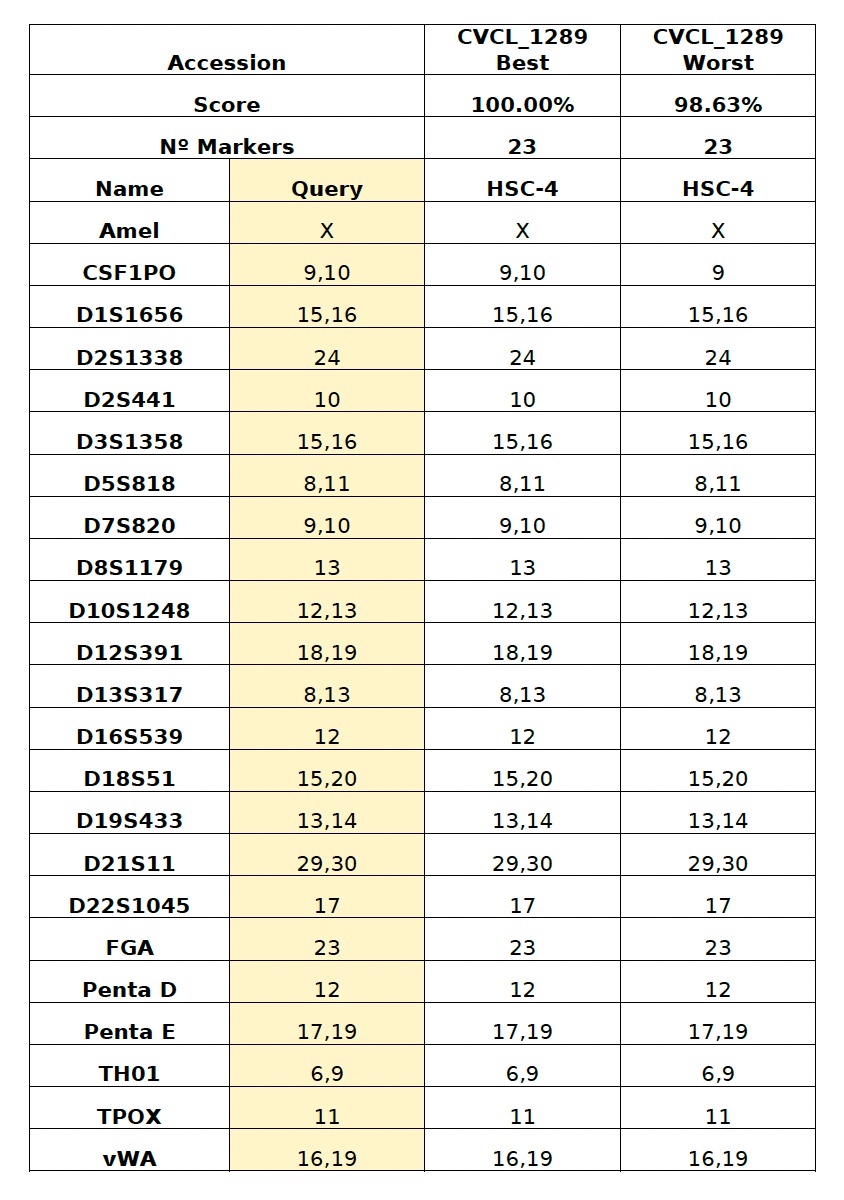


**Table S1:** Inhibitory effects of 2 mL and 5 mL PAM on normal human epidermal keratinocytes (HaCaT) and on three tumoral HNC cell lines (HSC3, HSC4, and A253) proliferation rates. Numbers represent the inhibition of proliferation over the cell inhibition of control cells at 24, 48, and 72 hours (expressed as 0% of inhibition). *p<0.05, **p<0.01, ***p<0.001 and ****p<0.0001

|  | 5mL vs. 2mL anti-tumor activity | | | | | | | | | |
| --- | --- | --- | --- | --- | --- | --- | --- | --- | --- | --- |
| HaCaT | **% inhibition of cell proliferation** | **24h** | | | **48h** | | | **72h** | | |
|  |  | **2 mL** | **5 mL** | **p-value** | **2 mL** | **5 mL** | **p-value** | **2 mL** | **5 mL** | **p-value** |
|  | **5'** | 0.79 | -3.72 | **ns** | 3.71 | -2.53 | **ns** | 5.07 | -1.86 | **ns** |
|  | **10'** | 6.98 | -2.36 | **ns** | 22.25 | 1.53 | **ns** | 19.57 | 6.28 | **ns** |
|  | **20'** | 31.36 | -1.81 | ******* | 54.25 | 10.84 | ****** | 61.92 | 61.92 | **ns** |
| HSC3 | **% inhibition of cell proliferation** | **24h** | | | **48h** | | | **72h** | | |
|  |  | **2 mL** | **5 mL** | **p-value** | **2 mL** | **5 mL** | **p-value** | **2 mL** | **5 mL** | **p-value** |
|  | **5'** | 74.67 | 20.32 | ******* | 79.88 | 19.99 | ****** | 99.35 | 71.14 | ****** |
|  | **10'** | 98.98 | 50.56 | ****** | 99.41 | 43.24 | ****** | 99.52 | 96.62 | ***** |
|  | **20'** | 99.13 | 76.11 | ******** | 99.46 | 71.26 | ******** | 99.47 | 99.45 | **ns** |
| HSC4 | **% inhibition of cell proliferation** | **24h** | | | **48h** | | | **72h** | | |
|  |  | **2 mL** | **5 mL** | **p-value** | **2 mL** | **5 mL** | **p-value** | **2 mL** | **5 mL** | **p-value** |
|  | **5'** | 60.94 | 18.80 | ****** | 66.90 | 10.55 | ******** | 77.03 | 12.83 | ***** |
|  | **10'** | 76.09 | 32.02 | ***** | 81.60 | 26.43 | ******* | 81.63 | 16.17 | ***** |
|  | **20'** | 74.69 | 55.11 | ***** | 80.46 | 54.46 | ******* | 79.72 | 65.16 | ***** |
| A253 | **% inhibition of cell proliferation** | **24h** | | | **48h** | | | **72h** | | |
|  |  | **2 mL** | **5 mL** | **p-value** | **2 mL** | **5 mL** | **p-value** | **2 mL** | **5 mL** | **p-value** |
|  | **5'** | 42.16 | 13.70 | **ns** | 43.08 | 22.12 | **ns** | 51.39 | 30.30 | **ns** |
|  | **10'** | 53.98 | 25.39 | **ns** | 59.31 | 33.05 | ***** | 60.08 | 48.36 | **ns** |
|  | **20'** | 63.43 | 44.24 | **ns** | 69.94 | 56.56 | **ns** | 68.35 | 58.51 | **ns** |

**Table S2.** Calibration parameter obtained during the validation procedure

|  | **Fluoride** | **Chlorite** | **Bromate** | **Chloride** | **Nitrite** | **Bromide** | **Chlorate** | **Nitrate** | **Phosphate** | **Sulphate** |
| --- | --- | --- | --- | --- | --- | --- | --- | --- | --- | --- |
| Slope | 0.1266 | 0.04038 | 0.02216 | 0.1045 | 0.2199 | 0.0003999 | 0.03462 | 0.04768 | 0.02101 | 0.06802 |
| **Intercept** | 0.003549 | -0.000029236 | -0.000046963 | 0.01663 | -0.0006067 | 0.0002064 | -0.0006250 | 0.003628 | -0.0007420 | 0.002854 |
| **Linearity** ^a^ | 0.0275-2.7 | 0.101-10 | 0.202-20 | 0.091-9 | 0.152-15 | 0.182-18 | 0.202-20 | 0.182-18 | 0.273-27 | 0.182-18 |
| **Weighting**  **factor** | 1/X^2^ | | | | | | | | | |
| **r^2^** | 0.9985 | 0.9973 | 0.9984 | 0.9930 | 0.9997 | 0.9989 | 0.9982 | 0.9994 | 0.9985 | 0.9976 |
| **LOD** ^a, b^ | 0.001 | 0.034 | 0.067 | 0.030 | 0.050 | 0.060 | 0.067 | 0.060 | 0.011 | 0.060 |
| **LOQ** ^a, b^ | 0.0275 | 0.101 | 0.202 | 0.091 | 0.152 | 0.182 | 0.202 | 0.182 | 0.273 | 0.182 |

^a^ Expressed as μg/mL. ^b^ For LOQ values, the precision and trueness must be within ±20% as required by ICH and CDER.

The validated precision (RSD%) values of the method were in the range 0.1 ÷ 4.0% for the ten anions studied at three quality control levels within the linearity range; while the trueness values were in the range -7.6 ÷ 11.8% at the same QC concentration level.

**Table S3:** Nitrite [NO_2_^-^] content in 5 and 2 mL of DMEM, McCoy’s 5A and RPMI media exposed for 5, 10 and 20 minutes to CAP treatment.

|  |  | **DMEM** | | **McCoy’s 5A** | | **RPMI** | |
| --- | --- | --- | --- | --- | --- | --- | --- |
| **Parameter** | **Unit** | **5 minutes**  **5 mL** | **5 minutes**  **2 mL** | **5 minutes**  **5 mL** | **5 minutes**  **2 mL** | **5 minutes**  **5 mL** | **5 minutes**  **2 mL** |
| Lambda_z | 1/minutes |  |  | 0,00037604 | 0,000493161 | 0,001030495 | - |
| t1/2 | minutes |  |  | 1843,280516 | 1405,517881 | 672,6350419 | - |
| Tmax | minutes | 2880 | 2880 | 5 | 5 | 1440 | 1440 |
| Cmax | μg/mL | 17,353 | 56,407 | 33,386 | 83,925 | 0,23923 | 6,31335 |
| T_infusion | minutes | 5 | 5 | 5 | 5 | 5 | 5 |
| Clast_obs/Cmax |  | 0,31924163 | 0,759072456 | 0,125393878 | 0,081928902 | 0,051414956 | 0,780596672 |
| AUC 0-t | μg/mL*minutes | 49403,72598 | 203446,8483 | 80194,448 | 204692,3984 | 365,184 | 12639,5136 |
| AUC 0-inf_obs | μg/mL*minutes |  |  | 91327,3066 | 218634,8581 | 377,120009 | - |
| AUC 0-t/0-inf_obs |  |  |  | 0,878099344 | 0,936229475 | 0,968349574 | - |
| AUMC 0-inf_obs | μg/mL*minutes^2 |  |  | 220508196,9 | 431604164,4 | 631561,5806 | - |
| MRT 0-inf_obs | minutes |  |  | 2411,982646 | 1971,586694 | 1672,196557 | - |
| Vz_obs | (J/sec)/(μg/mL) |  |  | 0,524128562 | 0,166941386 | 46,31769699 | - |
| Cl_obs | (J/sec)/(μg/mL)/minutes |  |  | 0,000197093 | 8,2329E-05 | 0,047730164 | - |
| Vss_obs | (J/sec)/(μg/mL) |  |  | 0,475385613 | 0,162318858 | 79,81421643 | - |
| **Parameter** | **Unit** | **10 minutes**  **5 mL** | **10 minutes**  **2 mL** | **10 minutes**  **5 mL** | **10 minutes**  **2 mL** | **10 minutes**  **5 mL** | **10 minutes**  **2 mL** |
| Lambda_z | 1/minutes |  |  | - | - | 0,00023287 | - |
| t1/2 | minutes |  |  | - | - | 2976,536953 | - |
| Tmax | minutes | 2880 | 4320 | 10 | 2880 | 1440 | 1440 |
| Cmax | μg/mL | 24,745 | 136,56 | 0,217 | 19,666 | 3,87725 | 6,10417 |
| T_infusion | minutes | 10 | 10 | 10 | 10 | 10 | 10 |
| Clast_obs/Cmax |  | 0,002719741 | 1 | 1 | 0,7163761 | 0,511367593 | 0,000750307 |
| AUC 0-t | μg/mL*minutes | 80721,2138 | 497794,308 | 1,085 | 71253,67672 | 8817,49559 | 11471,56555 |
| AUC 0-inf_obs | μg/mL*minutes |  |  | - | - | 17331,67552 | - |
| AUC 0-t/0-inf_obs |  |  |  | - | - | 0,508750327 | - |
| AUMC 0-inf_obs | μg/mL*minutes^2 |  |  | - | - | 89414242,28 | - |
| MRT 0-inf_obs | minutes |  |  | - | - | 5154,0074 | - |
| Vz_obs | (J/sec)/(μg/mL) |  |  | - | - | 4,459824539 | - |
| Cl_obs | (J/sec)/(μg/mL)/minutes |  |  | - | - | 0,001038561 | - |
| Vss_obs | (J/sec)/(μg/mL) |  |  | - | - | 5,352750406 | - |
|  |  |  |  |  |  |  |  |
| **Parameter** | **Unit** | **20 minutes**  **5 mL** | **20 minutes**  **2 mL** | **20 minutes**  **5 mL** | **20 minutes**  **2 mL** | **20 minutes**  **5 mL** | **20 minutes**  **2 mL** |
| Lambda_z | 1/minutes |  |  | 0,000107127 | - | - | 0,000790535 |
| t1/2 | minutes |  |  | 6470,349249 | - | - | 876,8074164 |
| Tmax | minutes | 4320 | 2880 | 20 | 2880 | 20 | 1440 |
| Cmax | μg/mL | 2,4898 | 318,88 | 140,242 | 202,441 | 0,031863 | 8,49071 |
| T_infusion | minutes | 20 | 20 | 20 | 20 | 20 | 20 |
| Clast_obs/Cmax |  | 1 | 0,909966131 | 0,582875316 | 0,109439787 | 0,686062204 | 0,102618038 |
| AUC 0-t | μg/mL*minutes | 4946,05 | 1295538,8 | 425498,781 | 634462,922 | 77,14252 | 12860,1313 |
| AUC 0-inf_obs | μg/mL*minutes |  |  | 1188554,097 | - | - | 13962,29592 |
| AUC 0-t/0-inf_obs |  |  |  | 0,357996983 | - | - | 0,921061362 |
| AUMC 0-inf_obs | μg/mL*minutes^2 |  |  | 11257755258 | - | - | 26379745,83 |
| MRT 0-inf_obs | minutes |  |  | 9461,807204 | - | - | 1879,355875 |
| Vz_obs | (J/sec)/(μg/mL) |  |  | 0,14136953 | - | - | 1,630776409 |
| Cl_obs | (J/sec)/(μg/mL)/minutes |  |  | 1,51445E-05 | - | - | 0,001289186 |
| Vss_obs | (J/sec)/(μg/mL) |  |  | 0,143293881 | - | - | 2,422839763 |

**Table S4:** Nitrate [NO_3_^-^] content in 5 and 2 mL of DMEM, McCoy’s 5A and RPMI media exposed for 5, 10 and 20 minutes to CAP treatment.

|  |  | **DMEM** | | **McCoy’s 5A** | | **RPMI** | |
| --- | --- | --- | --- | --- | --- | --- | --- |
| **Parameter** | **Unit** | **5 minutes**  **5 mL** | **5 minutes**  **2 mL** | **5 minutes**  **5 mL** | **5 minutes**  **2 mL** | **5 minutes**  **5 mL** | **5 minutes**  **2 mL** |
| Lambda_z | 1/minutes |  |  | 0,000710307 | 0,000511309 | - | 0,000627942 |
| t1/2 | minutes |  |  | 975,8415408 | 1355,631349 | - | 1103,83869 |
| Tmax | minutes | 2880 | 2880 | 5 | 5 | 1440 | 5 |
| Cmax | μg/mL | 4,3891 | 17,535 | 34,402 | 22,959 | 61,2305 | 64,0326 |
| T_infusion | minutes | 5 | 5 | 5 | 5 | 5 | 5 |
| Clast_obs/Cmax |  | 0,35155271 | 0,498967779 | 0,032817859 | 0,074699106 | 2,46609E-05 | 0,134248805 |
| AUC 0-t | μg/mL*minutes | 13512,21235 | 60017,4984 | 99895,00384 | 45133,96092 | 131787,6799 | 139138,3357 |
| AUC 0-inf_obs | μg/mL*minutes |  |  | 101484,4572 | 48488,12664 | - | 152827,9658 |
| AUC 0-t/0-inf_obs |  |  |  | 0,984337963 | 0,930825009 | - | 0,910424574 |
| AUMC 0-inf_obs | μg/mL*minutes^2 |  |  | 174268875,6 | 89746974,82 | - | 238633120,9 |
| MRT 0-inf_obs | minutes |  |  | 1714,697691 | 1848,406212 | - | 1558,949304 |
| Vz_obs | (J/sec)/(μg/mL) |  |  | 0,249704755 | 0,726027786 | - | 0,187564146 |
| Cl_obs | (J/sec)/(μg/mL)/minutes |  |  | 0,000177367 | 0,000371225 | - | 0,000117779 |
| Vss_obs | (J/sec)/(μg/mL) |  |  | 0,304130892 | 0,686174413 | - | 0,183612255 |
|  |  |  |  |  |  |  |  |
| **Parameter** | **Unit** | **10 minutes**  **5 mL** | **10 minutes**  **2 mL** | **10 minutes 5 mL** | **10 minutes**  **2 mL** | **10 minutes**  **5 mL** | **10 minutes**  **2 mL** |
| Lambda_z | 1/minutes |  | 0,000272539 | 5,2179E-06 | - | 0,000964782 | - |
| t1/2 | minutes |  | 2543,296029 | 132840,1365 | - | 718,4493167 | - |
| Tmax | minutes | 1440 | 10 | 10 | 4320 | 0 | 4320 |
| Cmax | μg/mL | 6,7402 | 3,8564 | 1,115 | 44,16468121 | 59,953 | 95,681 |
| T_infusion | minutes | 10 | 10 | 10 | 10 | 10 | 10 |
| Clast_obs/Cmax |  | 0,877703926 | 0,697619542 | 0,8666145 | 1 | 0,072019749 | 1 |
| AUC 0-t | μg/mL*minutes | 14677,63564 | 7928,363 | 4145,910805 | 85650,11815 | 18230,37764 | 314624,0105 |
| AUC 0-inf_obs | μg/mL*minutes |  | 17799,61329 | 189330,4269 | - | 22705,79144 | - |
| AUC 0-t/0-inf_obs |  |  | 0,445423329 | 0,021897752 | - | 0,802895494 | - |
| AUMC 0-inf_obs | μg/mL*minutes^2 |  | 92141684,51 | 36299264269 | - | 55080962,62 | - |
| MRT 0-inf_obs | minutes |  | 5171,611593 | 191719,4093 | - | 2420,855216 | - |
| Vz_obs | (J/sec)/(μg/mL) |  | 3,710508153 | 18,22031761 | - | 0,821687226 | - |
| Cl_obs | (J/sec)/(μg/mL)/minutes |  | 0,001011258 | 9,50719E-05 | - | 0,000792749 | - |
| Vss_obs | (J/sec)/(μg/mL) |  | 5,229833208 | 18,22712506 | - | 1,919131249 | - |
|  |  |  |  |  |  |  |  |
| **Parameter** | **Unit** | **20 minutes**  **5 mL** | **20 minutes**  **2 mL** | **20 minutes**  **5 mL** | **20 minutes**  **2 mL** | **20 minutes**  **5 mL** | **20 minutes**  **2 mL** |
| Lambda_z | 1/minutes | 1,25488E-05 |  | 0,000269739 | - | 0,00035628 | - |
| t1/2 | minutes | 55236,30531 |  | 2569,699371 | - | 1945,510526 | - |
| Tmax | minutes | 20 | 4320 | 20 | 2880 | 0 | 4320 |
| Cmax | μg/mL | 0,565688 | 22,85 | 99,226 | 58,961 | 59,953 | 95,666 |
| T_infusion | minutes | 20 | 20 | 20 | 20 | 20 | 20 |
| Clast_obs/Cmax |  | 0,859130828 | 1 | 0,242284679 | 0,104427179 | 0,324420796 | 1 |
| AUC 0-t | μg/mL*minutes | 1846,34536 | 53018,728 | 151816,3551 | 182647,9096 | 42318,8586 | 158401,2782 |
| AUC 0-inf_obs | μg/mL*minutes | 40575,26922 |  | 240943,1511 | - | 96910,69825 | - |
| AUC 0-t/0-inf_obs |  | 0,045504205 |  | 0,630092013 | - | 0,436678915 | - |
| AUMC 0-inf_obs | μg/mL*minutes^2 | 3257678352 |  | 936313939,1 | - | 475312543,1 | - |
| MRT 0-inf_obs | minutes | 80277,28867 |  | 3876,036747 | - | 4894,644706 | - |
| Vz_obs | (J/sec)/(μg/mL) | 35,35169612 |  | 0,27695855 | - | 0,521325425 | - |
| Cl_obs | (J/sec)/(μg/mL)/minutes | 0,00044362 |  | 7,47064E-05 | - | 0,000185738 | - |
| Vss_obs | (J/sec)/(μg/mL) | 35,61260896 |  | 0,289564825 | - | 0,909121555 | - |

**Table S5:** Sulphate [SO_4_^2-^] content in 5 and 2 mL of DMEM, McCoy’s 5A and RPMI media exposed for 5, 10 and 20 minutes to CAP treatment.

|  |  | **DMEM** | | **McCoy’s 5A** | | **RPMI** | |
| --- | --- | --- | --- | --- | --- | --- | --- |
| **Parameter** | **Unit** | **5 minutes**  **5 mL** | **5 minutes**  **2 mL** | **5 minutes**  **5 mL** | **5 minutes**  **2 mL** | **5 minutes**  **5 mL** | **5 minutes**  **2 mL** |
| Lambda_z | 1/minutes |  |  | 0,000658134 | 0,000459247 | 0,001605546 | - |
| t1/2 | minutes |  |  | 1053,200731 | 1509,312675 | 431,7206297 | - |
| Tmax | minutes | 4320 | 4320 | 5 | 5 | 0 | 4320 |
| Cmax | μg/mL | 149,68 | 147,88 | 130,912 | 127,448 | 38,348 | 61,34 |
| T_infusion | minutes | 5 | 5 | 5 | 5 | 5 | 5 |
| Clast_obs/Cmax |  | 1 | 1 | 0,040850342 | 0,088412141 | 0,008490664 | 1 |
| AUC 0-t | μg/mL*minutes | 524368,6102 | 538059,7797 | 347923,9341 | 301017,325 | 82606,27505 | 192211,2613 |
| AUC 0-inf_obs | μg/mL*minutes |  |  | 356049,6353 | 325553,0386 | 82809,07215 | - |
| AUC 0-t/0-inf_obs |  |  |  | 0,977178179 | 0,924633744 | 0,997551028 | - |
| AUMC 0-inf_obs | μg/mL*minutes^2 |  |  | 615251358,4 | 675655938 | 81316134,28 | - |
| MRT 0-inf_obs | minutes |  |  | 1725,493227 | 2072,909712 | 979,4713248 | - |
| Vz_obs | (J/sec)/(μg/mL) |  |  | 0,076815286 | 0,120393907 | 0,13538543 | - |
| Cl_obs | (J/sec)/(μg/mL)/minutes |  |  | 5,05547E-05 | 5,52905E-05 | 0,000217367 | - |
| Vss_obs | (J/sec)/(μg/mL) |  |  | 0,087231877 | 0,114612276 | 0,212905221 | - |
|  |  |  |  |  |  |  |  |
| **Parameter** | **Unit** | **10 minutes**  **5 mL** | **10 minutes**  **2 mL** | **10 minutes**  **5 mL** | **10 minutes**  **2 mL** | **10 minutes**  **5 mL** | **10 minutes**  **2 mL** |
| Lambda_z | 1/minutes |  |  | - | 9,1199E-05 | 0,000425576 | - |
| t1/2 | minutes |  |  | - | 7600,378849 | 1628,728668 | - |
| Tmax | minutes | 2880 | 4320 | 10 | 0 | 0 | 4320 |
| Cmax | μg/mL | 111,94 | 173,85 | 129,583 | 119,677242 | 38,348 | 65,252 |
| T_infusion | minutes | 10 | 10 | 10 | 10 | 10 | 10 |
| Clast_obs/Cmax |  | 0,008149008 | 1 | 0,979318722 | 0,362565638 | 0,069523834 | 1 |
| AUC 0-t | μg/mL*minutes | 360763,5566 | 605051,275 | 395781,6079 | 188999,6873 | 11322,92391 | 207061,1495 |
| AUC 0-inf_obs | μg/mL*minutes |  |  | - | 664781,6721 | 17587,61577 | - |
| AUC 0-t/0-inf_obs |  |  |  | - | 0,284303396 | 0,643800959 | - |
| AUMC 0-inf_obs | μg/mL*minutes^2 |  |  | - | 7708943246 | 62552785,01 | - |
| MRT 0-inf_obs | minutes |  |  | - | 11591,20304 | 3551,638137 | - |
| Vz_obs | (J/sec)/(μg/mL) |  |  | - | 0,296895248 | 2,404854555 | - |
| Cl_obs | (J/sec)/(μg/mL)/minutes |  |  | - | 2,70766E-05 | 0,001023447 | - |
| Vss_obs | (J/sec)/(μg/mL) |  |  | - | 0,31384989 | 3,634914891 | - |
|  |  |  |  |  |  |  |  |
| **Parameter** | **Unit** | **20 minutes**  **5 mL** | **20 minutes**  **2 mL** | **20 minutes 5 mL** | **20 minutes**  **2 mL** | **20 minutes**  **5 mL** | **20 minutes**  **2 mL** |
| Lambda_z | 1/minutes | 0,00055504 |  | 9,24632E-05 | 0,000414108 | 0,000332084 | - |
| t1/2 | minutes | 1248,823328 |  | 7496,461472 | 1673,833694 | 2087,266524 | - |
| Tmax | minutes | 0 | 2880 | 20 | 0 | 0 | 4320 |
| Cmax | μg/mL | 102,275 | 236,4 | 149,166 | 119,677242 | 38,348 | 64,642 |
| T_infusion | minutes | 20 | 20 | 20 | 20 | 20 | 20 |
| Clast_obs/Cmax |  | 0,050490345 | 0,978637902 | 0,623120139 | 0,066685044 | 0,353056222 | 1 |
| AUC 0-t | μg/mL*minutes | 10830,13124 | 956324,25 | 463742,8096 | 219846,0436 | 27151,4856 | 129935,8334 |
| AUC 0-inf_obs | μg/mL*minutes | 20133,78127 |  | 1468989,112 | 239118,0468 | 67921,32822 | - |
| AUC 0-t/0-inf_obs |  | 0,537908458 |  | 0,315688391 | 0,919403811 | 0,399749038 | - |
| AUMC 0-inf_obs | μg/mL*minutes^2 | 78740594,77 |  | 16138365754 | 559973799,8 | 356975318,1 | - |
| MRT 0-inf_obs | minutes | 3900,869683 |  | 10976,03497 | 2331,829934 | 5245,71757 | - |
| Vz_obs | (J/sec)/(μg/mL) | 1,610729827 |  | 0,132521023 | 0,181780375 | 0,798029199 | - |
| Cl_obs | (J/sec)/(μg/mL)/minutes | 0,00089402 |  | 1,22533E-05 | 7,52766E-05 | 0,000265012 | - |
| Vss_obs | (J/sec)/(μg/mL) | 3,487454908 |  | 0,134492916 | 0,175532292 | 1,390180651 | - |

**Table S6:** Phosphate [PO_4_^3-^] content in 5 and 2 mL of DMEM, McCoy’s 5A and RPMI media exposed for 5, 10 and 20 minutes to CAP treatment.

|  |  | **DMEM** | | **McCoy’s 5A** | | **RPMI** | |
| --- | --- | --- | --- | --- | --- | --- | --- |
| **Parameter** | **Unit** | **5 minutes**  **5 mL** | **5 minutes**  **2 mL** | **5 minutes**  **5 mL** | **5 minutes**  **2 mL** | **5 minutes**  **5 mL** | **5 minutes**  **2 mL** |
| Lambda_z | 1/minutes |  |  | 0,000932577 | 0,000493629 | 0,001374297 | - |
| t1/2 | minutes |  |  | 743,2599343 | 1404,186449 | 504,3648132 | - |
| Tmax | minutes | 2880 | 2880 | 5 | 5 | 0 | 4320 |
| Cmax | μg/mL | 189,52 | 180,61 | 954,685 | 935,185 | 795,637 | 1398,6 |
| T_infusion | minutes | 5 | 5 | 5 | 5 | 5 | 5 |
| Clast_obs/Cmax |  | 0,958051921 | 0,892863075 | 0,011376423 | 0,076166732 | 0,008664755 | 1 |
| AUC 0-t | μg/mL*minutes | 673223,5565 | 665256,6845 | 2439034,559 | 2192879,403 | 1716260,708 | 4120058,458 |
| AUC 0-inf_obs | μg/mL*minutes |  |  | 2450680,674 | 2337178,022 | 1721277,09 | - |
| AUC 0-t/0-inf_obs |  |  |  | 0,995247804 | 0,938259466 | 0,997085663 | - |
| AUMC 0-inf_obs | μg/mL*minutes^2 |  |  | 3874518810 | 4627433209 | 1701258009 | - |
| MRT 0-inf_obs | minutes |  |  | 1578,497007 | 1977,423295 | 985,8696346 | - |
| Vz_obs | (J/sec)/(μg/mL) |  |  | 0,007875915 | 0,015601991 | 0,007609236 | - |
| Cl_obs | (J/sec)/(μg/mL)/minutes |  |  | 7,3449E-06 | 7,7016E-06 | 1,04574E-05 | - |
| Vss_obs | (J/sec)/(μg/mL) |  |  | 0,0115939 | 0,015229315 | 0,010309586 | - |
|  |  |  |  |  |  |  |  |
| **Parameter** | **Unit** | **10 minutes**  **5 mL** | **10 minutes**  **2 mL** | **10 minutes**  **5 mL** | **10 minutes**  **2 mL** | **10 minutes**  **5 mL** | **10 minutes**  **2 mL** |
| Lambda_z | 1/minutes |  |  | - | 6,27413E-05 | 0,000445112 | - |
| t1/2 | minutes |  |  | - | 11047,7087 | 1557,243616 | - |
| Tmax | minutes | 2880 | 2880 | 10 | 0 | 0 | 4320 |
| Cmax | μg/mL | 141,01 | 175 | 951,873 | 861,3204188 | 795,637 | 1339,3 |
| T_infusion | minutes | 10 | 10 | 10 | 10 | 10 | 10 |
| Clast_obs/Cmax |  | 0,018477413 | 0,882 | 0,950241657 | 0,389241207 | 0,058496525 | 1 |
| AUC 0-t | μg/mL*minutes | 462941,651 | 658567,635 | 3146647,738 | 1372789,225 | 199651,9958 | 2723703,72 |
| AUC 0-inf_obs | μg/mL*minutes |  |  | - | 6716344,505 | 304214,5395 | - |
| AUC 0-t/0-inf_obs |  |  |  | - | 0,204395296 | 0,65628683 | - |
| AUMC 0-inf_obs | μg/mL*minutes^2 |  |  | - | 1,11521E+11 | 1051661955 | - |
| MRT 0-inf_obs | minutes |  |  | - | 16599,38115 | 3451,974662 | - |
| Vz_obs | (J/sec)/(μg/mL) |  |  | - | 0,042715579 | 0,132930193 | - |
| Cl_obs | (J/sec)/(μg/mL)/minutes |  |  | - | 2,68003E-06 | 5,91688E-05 | - |
| Vss_obs | (J/sec)/(μg/mL) |  |  | - | 0,044486828 | 0,204249093 | - |
|  |  |  |  |  |  |  |  |
| **Parameter** | **Unit** | **20 minutes**  **5 mL** | **20 minutes**  **2 mL** | **20 minutes**  **5 mL** | **20 minutes**  **2 mL** | **20 minutes**  **5 mL** | **20 minutes**  **2 mL** |
| Lambda_z | 1/minutes | 0,000764839 | 6,50169E-05 | 0,000115389 | 0,000429749 | 0,000335634 | - |
| t1/2 | minutes | 906,2655343 | 10661,03085 | 6007,039997 | 1612,910464 | 2065,187267 | - |
| Tmax | minutes | 0 | 20 | 20 | 0 | 0 | 4320 |
| Cmax | μg/mL | 135,333 | 239,59 | 1104,267 | 861,3204188 | 795,637 | 1384,4 |
| T_infusion | minutes | 20 | 20 | 20 | 20 | 20 | 20 |
| Clast_obs/Cmax |  | 0,044258237 | 0,741516758 | 0,575308976 | 0,059406592 | 0,323966834 | 1 |
| AUC 0-t | μg/mL*minutes | 16843,42548 | 894736,13 | 3421143,582 | 1550080,004 | 496900,22 | 2667748,009 |
| AUC 0-inf_obs | μg/mL*minutes | 24674,6166 | 3627256,429 | 8926815,22 | 1669145,021 | 1264879,496 | - |
| AUC 0-t/0-inf_obs |  | 0,682621568 | 0,246670217 | 0,383243463 | 0,928667063 | 0,39284392 | - |
| AUMC 0-inf_obs | μg/mL*minutes^2 | 73173615,83 | 55663102110 | 78162716509 | 3826282358 | 6693755588 | - |
| MRT 0-inf_obs | minutes | 2955,542161 | 15335,78633 | 8745,946503 | 2282,360646 | 5282,010512 | - |
| Vz_obs | (J/sec)/(μg/mL) | 0,953788529 | 0,076325206 | 0,017474752 | 0,025093615 | 0,042399168 | - |
| Cl_obs | (J/sec)/(μg/mL)/minutes | 0,000729495 | 4,96243E-06 | 2,0164E-06 | 1,0784E-05 | 1,42306E-05 | - |
| Vss_obs | (J/sec)/(μg/mL) | 2,156052099 | 0,07610274 | 0,017635297 | 0,024612895 | 0,075166203 | - |

**Table S7:** Effects of PAM in the induction of apoptosis of normal cells (HaCaT) and three tumor head and neck cancer (HNC) cell lines (A253, HSC3 and HSC4). Numbers represent the percentage of early and late apoptosis and necrosis at 24 h.

|  |  | Early apoptosis | Late apoptosis | Necrosis |
| --- | --- | --- | --- | --- |
| HaCaT | CTRL | 2.85±1.88 | 9.96±1.96 | 1.92±0.99 |
|  | 2ml 5min | 1.64±1.38 | 10.62±2.76 | 3.07±1.71 |
|  | 5ml 5 min | 1.2±0.82 | 9.45±1.52 | 2.54±1.00 |
|  | 5ml 10 min | 1.57±0.68 | 8.56±1.43 | 1.80±0.85 |
|  | 5ml 20 min | 2.76±2.00 | 9.33±1.54 | 1.70±1.26 |
| A253 | CTRL | 2.36±1.35 | 17.60±11.89 | 9.67±8.13 |
|  | 2ml 5min | 19.46±4.11 | 11.76±3.94 | 3.02±1.18 |
|  | 5ml 5 min | 3.92±1.97 | 8.22±0.89 | 2.46±0.17 |
|  | 5ml 10 min | 10.74±5.41 | 9.97±3.17 | 2.62±0.60 |
|  | 5ml 20 min | 18.20±5.01 | 13.59±6.40 | 2.78±0.92 |
| HSC3 | CTRL | 0.74±0.10 | 5.31±2.38 | 1.26±0.30 |
|  | 2ml 5min | 6.30±1.34 | 26.75±16.35 | 1.63±0.28 |
|  | 5ml 5 min | 1.73±0.76 | 7.88±2.39 | 1.52±0.19 |
|  | 5ml 10 min | 3.96±0.36 | 11.82±1.98 | 1.546±1.062 |
|  | 5ml 20 min | 8.20±3.97 | 25.90±19.32 | 2.06±0.72 |
| HSC4 | CTRL | 3.69±1.74 | 9.386±1.90 | 0.96±0.40 |
|  | 2ml 5min | 9.14±2.66 | 41.83±7.83 | 1.80±0.46 |
|  | 5ml 5 min | 9.68±6.37 | 18.89±6.39 | 1.30±0.25 |
|  | 5ml 10 min | 14.14±7.21 | 33.85±11.09 | 2.06±1.04 |
|  | 5ml 20 min | 7.88±2.26 | 64.86±15.89 | 6.12±1.99 |

**Table S8:** Effects of PAM on cell cycle of normal cells (HaCaT) and tumoral HSC4 cell line. Numbers represent the percentage of G1, S and G2/M phases at 24 hours.

|  |  | G_0_/G_1_ | S | G_2_/M |  |
| --- | --- | --- | --- | --- | --- |
| HaCaT | CTRL | 36.55±3.05 | 51.53±2.06 | 11.92±5.11 |  |
|  | 2ml 5min | 38.99±0.03 | 53.75±5.28 | 7.27±5.31 |  |
|  | 5ml 5 min | 34.85±1.51 | 51.23±4.24 | 13.94±5.75 |  |
|  | 5ml 10 min | 36.77±3.83 | 54.59±4.21 | 8.65±0.38 |  |
|  | 5ml 20 min | 45.89±1.06 | 44.64±5.36 | 9.47±4.30 |  |
| HSC4 | CTRL | 49.22±7.41 | 41.16±4.34 | 9.61±3.07 |  |
|  | 2ml 5min | 27.89±3.54 | 56.54±3.23 | 15.56±5.76 |  |
|  | 5ml 5 min | 49.97±7.92 | 40.96±5.58 | 9.06±3.28 |  |
|  | 5ml 10 min | 49.93±6.99 | 42.22±9.25 | 7.84±7.64 |  |
|  | 5ml 20 min | 27.69±2.04 | 57.73±8.86 | 14.57±9.44 |  |
